# Energy from waste biomass: an LCA study on a biofuel cell at early design stage

**DOI:** 10.1007/s11356-024-34068-1

**Published:** 2024-06-26

**Authors:** Eleonora Rossi, Daniele Cespi, Irene Maggiore, Leonardo Setti, Fabrizio Passarini

**Affiliations:** 1 Industrial Chemistry Deparment “Toso Montanari”, Alma Mater - Università di Bologna, Via Piero Gobetti, 85, 40136 Bologna, BO Italy; 2 Interdepartmental Center for Industrial Research Renewable Sources, Environment, Sea and Energy, University of Bologna, Via Angherà 22, 47922 Rimini, RN Italy

**Keywords:** Biomass fuel cell, Life cycle assessment, Sustainable waste treatment, Biomass valorization, Ex-ante LCA, Ecodesign

## Abstract

Diversifying energy sources and managing waste biomass are two pressing contemporary issues. The new technology proposed in this study aims to address both by converting waste biomass into energy and fertilizer through the use of a biofuel cell (BFC). The purpose of this study is to assess the environmental impacts associated with this innovative technology through a Life Cycle Assessment (LCA). To achieve the goal, the production and use of the cell were modelled, considering both laboratory-scale operations and industrial-scale approximations. The study explored alternative scenarios, such as sensitivity analyses involving different acids and bases, renewable energy sources, and heat recovery. Comparisons with conventional biomass waste treatments (anaerobic digestion and composting) demonstrated that the BFC technology remains competitive. To further improve the BFC’s environmental footprint, efforts should focus on reducing energy requirements and enhancing nutrient recovery during scale-up. These insights are crucial for advancing sustainable waste treatment technologies and maximizing the potential of discarded biomass in an environmentally friendly manner.

**Supplementary Information:**

The online version contains supplementary material available at 10.1007/s11356-024-34068-1.

## Introduction

The increasing global demand for energy, coupled with concerns over climate change and environmental degradation, has driven a shift toward sustainable and alternative energy sources. Vegetable biomass, deemed renewable and plentiful, represents a significant portion of global waste (Valerio et al. 2023): Estimates suggest the dumped food and green scraps from pruning activities categories contribute nearly half the mass of generated waste globally (Kaza et al. 2018). This resource has emerged as a promising solution for addressing these challenges.

Specifically, the fraction of industrial/agricultural waste in Europe, comprising 46% of agricultural activities (about 442 Mt) (Camia et al. 2018), has attracted attention for its potential in energy recovery and waste reduction (Kaza et al. 2018). Often, this waste is disposed of in landfills or incinerated without energy recovery, leading to environmental pollution through the release of pollutant substances into the atmosphere and groundwater, as well as the production of methane, a potent greenhouse gas (Bakkaloglu et al. 2022; Lee et al. 2023). To address these issues, the European Union has introduced directives and strategies aimed at reducing food waste generation throughout the supply chain (European Parliament 2008; European Commission 2020). As such, it becomes imperative to explore viable methods of harnessing the energy potential of biomass waste while mitigating its adverse environmental implications (Commission of the European Communities 2008).

Anaerobic digestion (AD) is a leading technology for processing agri-food waste due to its low carbon footprint, biogas generation, economic benefits, and reduced odour emissions (Cremiato et al. 2018; Lin et al. 2018; Valenti et al. 2020). However, achieving net-zero CO_2_ equivalent emissions with current commercial technologies remains a challenge due to fugitive methane emissions (Bakkaloglu et al. 2022).

In order to find an alternative way to valorise the waste from the agri-food sector and investigate a process to produce renewable energy, an innovative BFC (biofuel cell) system fed by waste biomass was implemented. This type of fuel cell can generate electricity by oxidizing organic substances, which will be analysed in detail in the “Overview of existing BFC technology”. Further details about the technology cannot be shared since the patent is pending (Setti and Maggiore 2022). Doing so, there is an opportunity to synergistically address two main environmental challenges: the effective management of waste streams, avoiding obsolete/superseded technologies and further transportation, and the generation of more sustainable energy. A BFC converts the chemical energy in the organic matter into electricity through an electrochemical process. This technology harnesses the energy-rich components present in waste biomass to generate power without the emissions associated with conventional combustion methods (Katz and Bollella 2021).

To assess the potential environmental impacts of this new technology, an analysis of the set-up process was performed using Life Cycle Assessment (LCA) methodology approach that is also explicated in the Ecodesign Directive 2009/125/EC by the European Commission for energy-related products (European Parliament 2009).

Although this type of analysis is usually more applied to advanced technologies (i.e. higher technology readiness level — TRL), conducting a LCA study on an early design stage appliance is crucial to identify the potential environmental hotspots and sustainability challenges before the product/process is upscaled for market. Early implementation of these tools allows for a better understanding of the environmental implications of design choices, helping to prevent avoidable burdens, reduce costs, prevent regrettable investments and substitutions, and anticipate changes in environmental regulations (Cucurachi et al. 2018). The adoption of the LCA methodology during early design stages is also one of the pillars of the new sustainable by design (SSbD) framework for chemicals and materials developed by the European Commission (Caldeira et al. 2022).

Applying LCA during early design stages has the potential to drive the development of emerging technologies with improved environmental performance, as it identifies inefficiencies and allows for comparison with existing alternatives (Moni et al. 2020). This application of the LCA, so-called *ex ante* (Cucurachi et al. 2018) or *at early stage* (Patel et al. 2012, 2013; Cespi et al. 2016), enables testing alternative policy interventions, verifying claims of environmental sustainability, and supporting early design improvements and informed investments.

In particular, it is crucial to assess and verify, early in their development, that emerging biobased technologies and products genuinely reduce environmental burdens. In fact, bioeconomy-related advancements have the potential to greatly impact the shift towards a more circular and decarbonized economy. This validation is necessary to steer investments and technology implementation in the direction of a sustainable economy (Cucurachi et al. 2022).

Precisely for these reasons and to support the development of this process from the perspective of eco-design, the environmental impacts have been analysed, starting from the production of the cell to its use and the recovery of the output co-products (electricity and soil conditioner). In addition to studying the potential impacts of the system on a laboratory scale, additional scenarios were created to simulate industrial upscaling and study the optimization of the process from an environmental perspective. After that, a sensitivity analysis was applied by investigating scenarios with different reagents and energy supplies. Finally, to assess whether this technology could be promising, the process under consideration was compared with the two main traditional treatments of waste biomass: composting and anaerobic digestion.

## Biofuel cell

### Overview of existing BFC technology

Fuel cells (FCs), which directly convert fuel chemical energy into electricity through electrochemical reactions (Encyclopædia Britannica 2020), offer a versatile alternative for energy supply diversification and they find applications ranging from microelectronics to large-scale power generation (Tellez-Cruz et al. 2021). Each cell in a FC system has a pair of electrodes: anode, which supplies electrons, and cathode, which absorbs electrons. The electrolyte is critical for conducting electrons between the two electrodes. The voltage per unit cell depends on energy level differences (Ferriday and Middleton 2021), and electric power output relies on chemical activity and fuel supply.

While most organic substrates generate energy through combustion, biofuel cells (BFCs) offer an alternative by oxidizing organic substances with an oxidizer to convert chemical energy into electricity. Unlike conventional fuel cells using inorganic catalysts, BFCs employ bioelectrocatalysts like redox enzymes or whole biological cells. Some BFCs, known as “abiotic” biocells, use inorganic catalysts to oxidize bioorganic materials like glucose. This kind of fuel cell operate under conditions compatible with biological systems, presenting a unique approach to energy production and biomass waste processing (Katz and Bollella 2021).

Biomass-powered fuel cells (BFCs) can be classified into Indirect biomass fuel cells (IDBFC) and direct biomass fuel cells (DBFC) depending respectively on the presence or the lack of pre-conversion technology to which biomass is subjected before its usage as a fuel. Pre-conversion techniques are aimed at disassembling the lignocellulosic structure of biomass to simple components (cellulose, hemicellulose, and lignin) to obtain more usable fuels such as fermentable sugars, syngas, biogas, or biochar (Liu et al. 2020).

IDBFCs have been largely studied in recent years. They include the biotic microbial fuel cell (MFC) and enzymatic fuel cell (EFC), as well as the abiotic solid oxide fuel cell (SOFC) and direct carbon fuel cell (DCFC) (Zhao et al. 2017). MFCs can work at ambient temperature but suffer from feedstock contamination risk due to the operative pH close to neutral (Oliveira et al. 2013), membrane biofouling and short lifetime of the electrode materials (Do et al. 2018), instability of anodic biofilm responsible for the electrical generation (Kim et al. 2015), stabilization of output stream, and scale-up difficulties related to biofuel cell configuration (Oliveira et al. 2013). The abiotic IDBFCs find application on a commercial scale but operate at high temperatures (> 600 °C) and require high energy–demanding processes to pretreat biomass (Gür 2013).

DBFCs instead can work at a lower temperature (*T* < 100 °C) (Giddey et al. 2012) and that makes them more suitable for distributed applications, but generally require biomass in purified form, like starch or cellulose (Antolini 2021). Moreover, their efficiency for directly converting biomass into electrical energy is strictly related to the catalyst selectivity and stability. In the most studied proton exchange membrane fuel cell (PEMFC) and alkaline fuel cells, whether they with anionic (AAEMFC), cationic (ACEMFC), or without exchange membrane (AFC), catalysts are precious or transition metal, and composites, mostly deposited on a support (Fujiwara et al. 2007; Tung et al. 2012; Antolini 2021); they are susceptible to poisoning from oxidation byproducts and generally are unable to electrochemically oxidate large molecules (biomass polymers) completely at ambient temperature. Moreover, as developed so far, direct biomass fuel cells are affected by high internal resistance due to the implicit constraint of the structural configuration, potentially limiting the power output (Netwall et al. 2013).

### Innovative technology analysed

The BFC object of the present study has been designed and realized to make the processing of biomass for direct energy production efficient, easy to manage, and feasible at a local scale (Setti and Maggiore 2022). It is an abiotic system that relies on the alkaline degradation of carbohydrates for the anode electrolyte generation under mild conditions (*T* < 100 °C) and works at ambient temperature through the oxidation of the reducing sugars at the anode, paired with the reduction of iron (III) species at the cathode. The two half-chambers are separated by an ion-conducting membrane that, at the early stage of technology, consists of an agar and potassium chloride salt bridge. The catalyst for the sugar’s oxidation is an inorganic base it is a low-cost reagent, active at low temperatures; it is non-selective and thus capable of oxidizing various organic substrates and high molecular weight polymers (Xu and Sun 2016); it allows to overcome feedstock sanitization issue because the high pH (14) ensures the sterility of the organic stream.

This adds economic value to the waste generated by the whole process.

The novel BFC technology is aimed at converting waste from the agribusiness sector into energy and fertilizer. At the end of the fuel cell operations, the reduced catholyte can be easily regenerated by bubbling molecular oxygen and the exhausted biofuel neutralized with inorganic acid (H_3_PO_4_) to give a potential bio-fertilizer.

Some potential advantages can be identified compared to traditional treatment techniques (e.g. composting and anaerobic digestion), such as (i) lower initial investment costs, (ii) the possibility of varying the number of cells (and thus the overall capacity) depending on the input stream, (iii) the avoided transportation of waste to pre-treatment and treatment plants (on-site application), and (iv) the direct production of electricity and soil conditioner.

The BFC, in fact, is made of acrylonitrile butadiene styrene (ABS) and is produced in a few hours (7 h in TRL 4) through additive manufacturing. The BFC is small in size (63 mm base diameter, 70 mm height) and can be used as an on-site battery. In addition, a great intrinsic advantage of the BFC is given by the unique 3D-printed design. The fuel cell consists of a single body made of recyclable plastic (ABS), with environmental and economic benefits. It has a tubular shape with a coaxial and concentric arrangement of the half chambers, which are predisposed to work by continuously feeding electrolytes, and it uses organic-based electrodes (activated carbon felt and graphite rods). Concerning the common planar configuration, the tubular design enables a higher surface-to-volume ratio (Percin et al. 2020) and allows to arrange more BFCs in height. This enables the stacked system to reach a higher power output per covered surface (31 cm^2^).

The reagents used (NaOH and H_3_PO_4_) are quite cheap (respectively around $0.34/kg and $1.46/kg in Europe in October 2023) (Business Analytiq 2023) and common in the food industry (Kurt and Bittner 2006)(Matrakova et al. 2022), the sector for which this process was designed. In addition, this BFC can work with biomass with a low carbon oxygen demand (COD). Low COD biomass often includes various waste materials, which might otherwise be challenging to dispose of or utilize effectively. Thus, the ability broadens the range of feedstocks that can be used efficiently.

This cell was mainly designed for energy valorization of agro-food waste by continuous power generation. Thus, the major fields of application are in the treatment of wastewater from biorefinery plants (for chemicals recovery and upgrading of biomass) and in anaerobic fermentation plants to implement their efficiency in terms of material and energy recovery.

The added value of this BFC system is that it can process even small volumes of feedstock and thus be used at the same site where the biomass comes from. This can be an advantage for small businesses in the agricultural sector that would otherwise be forced to delegate the management of their waste. Its ability to join multiple cells in stacks then allows for variable volumes to be processed. Thus, there will be no problems of over and under sizing and no very high initial investment will be required. Downstream of the process, energy and soil conditioner will be produced, and both could be sold to farmers by favouring a circular market.

The following data about BFC preparation are available:

1. 3D cell printing: The initial step involves printing the cell structure using a fused deposition modelling (FDM) 3D printer, taking approximately 7 h to complete.
2. Preparation of salt bridge: For the cell-up, a salt bridge was crafted using water, potassium chloride, and agar. The salt was melted on a hot plate, creating a solution poured into the printed cell. To accelerate the formation of the agar gel, the cell was then placed in the freezer.
3. Alkaline hydrolysis reaction: To pre-treat the biomass for the reaction within the BFC, hydrolysis was required, after which the anolyte acting as biofuel was generated. Glucose was selected as a model molecule on a lab scale; a heating magnetic stirrer was employed to aid in its dissolution.
4. Felt saturation: felt, industrially manufactured from polyacrylonitrile (PAN) precursor, serves as an activated carbon fabric. The carbon-felt electrodes play a crucial role as they furnish the necessary surface area for the reaction to take place (Banerjee et al. 2019).
5. Cell feeding and reaction: During this phase, the solution was introduced into the cell using a peristaltic pump, while a heater maintained the temperature constant. The electrochemical reaction takes place with anolyte recirculation for as long as the cell operation.
6. Cell output: Energy was generated, and the exhausted biofuel left the cell. The effluent departed from the anode was sent to the neutralization step. The catholyte, having undergone reaction in the laboratory phase, was discarded on the lab configuration, although there is potential for regeneration by introducing gaseous O_2_ into the solution.
7. Neutralization (downstream process): Anolyte was neutralized.
8. Downstream process: The output from neutralization was a liquid soil conditioner; its specific characteristics should be further studied in subsequent analyses.

**Fig. 1:**
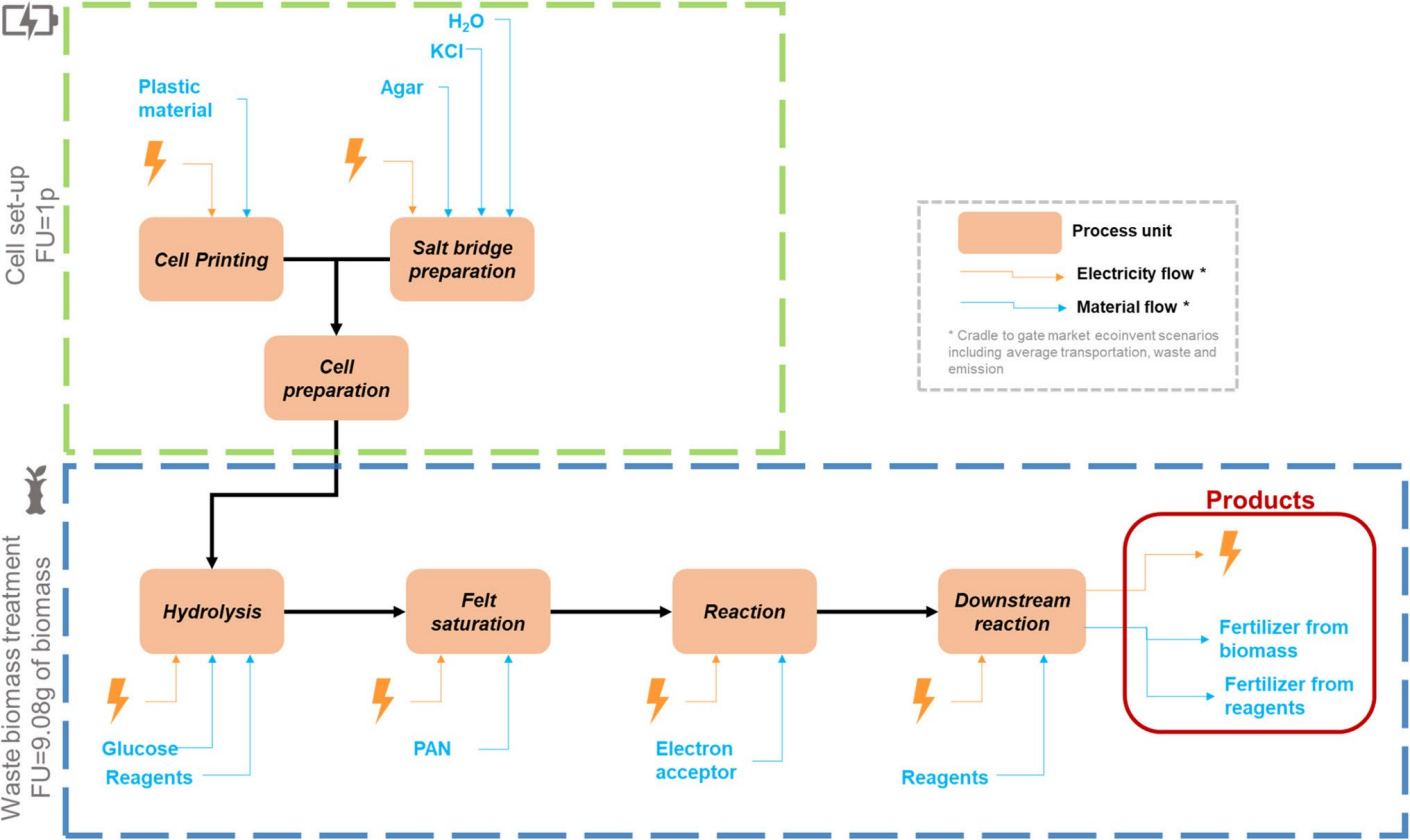
System boundaries and functional units of the studied process, subdivided into production (green dashed line) and use (blue dashed line) of the BFC

System configuration is reported later in the text (Fig. 1).

## Material and methods

LCA is an analytical and internationally standardized methodology (ISO 2006a)(ISO 2006b) useful for quantifying the potential environmental impacts of a process, product, or system throughout the entire life cycle or a part of it. A cradle-to-grave LCA considers the entire chain from resource extraction, through production, use, and recycling, to waste disposal, determining all emissions and resources consumed directly or indirectly (Curran 2012). According to the ISO series, the Life Cycle Assessment (LCA) framework involves defining study objectives and system boundaries, conducting inventory analysis (LCI, Life Cycle Inventory) to quantify inputs and outputs, assessing environmental impacts (LCIA, Life Cycle Impact Assessment), and interpreting results to inform decision-making.

### Goal and scope definition

As depicted in Fig. 1, the analysis was divided into two stages. In the first stage, the LCI for cell production was compiled using 1 unit (1 cell unit = 1p) as the functional unit (FU). The material and energy flows for infrastructure assembly were then incorporated into the cell usage stage to identify the manufacturing impacts across the cumulative cradle-to-gate life cycle. This second stage of the analysis entails evaluating the cell’s usage to fulfill its function, namely, treating biowaste to co-produce energy and soil conditioner. Consequently, the function was expressed by adjusting the FU and specifying a fixed amount of biomass treatable in one usage cycle, equivalent to 9.08 g of waste biomass. Thus, cell manufacturing (1p) was considered within its usage phase. The impacts were distributed based on the various lifetime scenarios considered. Assuming a lifespan of 10 cycles for the technology (representing the current scenario at the lab scale), the burdens associated with cell manufacturing (1p) were divided by 10.

In line with the literature (Cucurachi et al. 2022)*,* the choice of the functional unit for simulating the BFC use was made following the guidelines of the Joint Research Center (Manfredi et al. 2011). Since the process has two outputs, it was decided to adopt a feedstock-based approach. Given that the feedstock utilized is sourced from waste, the usage of the treated mass as FU is preferable rather than amount of land used, better when the feedstock derives from dedicated crops (Cucurachi et al. 2022). In addition, the ISO standard (ISO 2006b) recommends that allocation should be avoided whenever possible.

In this study, the allocation was avoided (in line with ISO standards) through a system boundaries expansion. As written above, the functional unit is represented by the quantity of biomass to be treated by the system, since the main aim of the technology is to treat the waste biomass to co-produce soil conditioner and energy, both considered avoided products from traditional manufacturing processes.

In this step, the goals of the study were also set. First is to assess the environmental impacts of the production and use of a waste BFC at the laboratory stage. Then, the second goal was to create scenarios for the optimization of the BFC after identifying its environmental hotspots. After the optimization, a further comparison was done to counterpose the studied technology with more mature alternatives used for biomass disposal and assess the absolute value of the impacts. Finally, the study aimed to evaluate the environmental feasibility of obtaining energy from biomass without any combustion step.

### Life Cycle Inventory

Inventory analysis is one of the most time-consuming and resource-intensive parts of a common LCA. The difficulties are even greater when chemicals are involved. One reason is that only a small portion of the huge number of chemicals on the world market is represented in the current LCI databases (Maranghi 2020).

In the study, primary data regarding cell production and its use were obtained directly from the developer’s team. This dataset represents mostly the foreground information, data having the highest quality in terms of technological, temporal, and geographic correlation. This allowed for a comprehensive inventory with relatively low degrees of uncertainty. Nonetheless, some approximations were necessary to complete the inventory, particularly for energy consumption and soil conditioner.

It was possible to have the exact energy consumption figure for only one stream (additive manufacturing), in other cases the electricity needed was calculated by considering instrument power (kW) per the time of use (h) (Giménez et al. 2015). When feasible, the energy consumption was allocated per the volume occupied by the reagent within the instrumentation (such as, for example, during heating in the stove). The powers (*P*) of the instruments were calculated by considering the performance of the equipment relative to the maximum value allowable. In the case the stove was working at a temperature (*T*) below the upper bound, the *P*_max_ value was multiplied by a corrector factor calculated as follow: ∆*T*_working_/∆*T*_max_. All allocations are made explicit in the spreadsheet detail found in the SI_2.

An example of the calculation is given in Table 1, where the data for estimating the energy consumption related to the heater are shown. Knowing the device’s parameters from the data sheet (maximum power, temperature and volume), the consumption was allocated on the basis of the volume occupied by the sample (0.5 l) and the temperature of use (30°). The power consumption value is then calculated as follows: *P*_max_ × time × (Δ*T*/Δ*T*_max_) × (OccVol/Vol).

**Table 1:** Calculation for heater energy consumption

Heater		
*P*_max_	1600	W
*T*_max_	300	°C
Volume	122.2	l
Time	0.5	h
*T*	30	°C
Δ*T*/Δ*T*_max_	3.57E − 02	
Occupied Vol	0.5	l
OccVol/Vol	4.09E − 03	
Energy consumption	0.12	Wh

The power output of the 3D printer was 360 W, and from an initial calculation based on assumptions, the resulting power output would have been 2520 Wh, which is a very high-power consumption. For this reason, it was decided to make a timely measurement of this power consumption using an instrument that could detect the printer’s power consumption in real time. The figure thus obtained, 1270 Wh, is about half of what was initially assumed and is to be considered with a high-quality level. In this study, life cycle impacts related to the infrastructure of the main appliances used at lab scale (e.g. freezer) were not taken into account since out of scope and negligible.

The input matter fluxes are all accurate because primary data could be obtained from laboratory-scale measures, only a few approximations were necessary. On the other hand, the output inventory has a lower degree of accuracy because laboratory tests have not yet been conducted. The usage of glucose as a model molecule did not allow exact information regarding the future output composition. Therefore, an approximation was carried out to have a representation of a fertilizer containing the nutrients present in an average waste biomass stream. To create a model more similar to reality, the 9.08 g of glucose was replaced with the same amount of a standard biomass found in the ecoinvent database (Wernet et al. 2016). Those values were also used to estimate the nutrient amounts. The elements considered were nitrogen, potassium, and phosphorus. To model the final soil conditioner composition, all the nutrient inputs were calculated and the corresponding fertilizer outputs were estimated by assuming them in different forms. A 100% nutrient recovery was assumed, without considering energy consumption for the associated fertilizer purification steps. N, K, and P were transformed into the corresponding fertilizers as: elemental nitrogen, potassium oxide, and phosphorus dioxide. The inventory is given on SI (Tables S1–5).

Following the establishment of the baseline scenario using laboratory data, various other scenarios were formulated. These included scenarios for three distinct cell life expectancies (scenario 1), alterations in reagents (scenario 2), increased percentages of renewable energy sources (scenario 3), and an optimized scenario simulating industrial-scale energy consumption (scenario 4).

According to the literature, the average service life of an industrial fuel cell is 5000 h with an efficiency loss of 10% (Hua et al. 2020). Initially, each cycle was approximated to one hour. An estimated efficiency loss of 2.5% per 1000 h was factored in. According to this estimation and based on the duration of one cycle, the equivalent of 5000 h corresponds to approximately 4750 cycles (c).

Another noteworthy factor concerning the cell’s usage expectation is the energy break-even point, indicating the number of cycles needed to recover the energy invested in producing the cell. With an energy consumption of 1270 Wh for production and an energy output of 5.4 Wh, it takes approximately 237c to recuperate the energy spent. This number of cycles was therefore taken as a reference, in addition to 10, which represents the use of a cell in the experimental laboratory phase, and 4750 cycles, which represents the average life of an upscaled BFC.

### Sensitivity analysis

Across the three cases where cycles and cell allocation were adjusted (scenario 1), there were changes made to the PAN parameter. Specifically, in the 237c and 4750c scenarios, the infrastructure’s felt aspect was factored in. Additionally, its impact was distributed proportionally by the number of cycles. This decision aimed to align the scenarios more closely with an industrial setting.

In the case of scenario 2 (i.e. reagent changes), phosphoric acid was substituted with nitric acid, and sodium hydroxide was replaced by potassium hydroxide. As these substituted reagents were not tested in the laboratory, their quantities were determined through stoichiometric calculations at the same pH level. Although H_3_PO_4_ is a triprotic acid in first approximation, it has been considered the Ka1 of orthophosphoric acid. The pH of the 14.56 M phosphoric acid solution was then calculated, followed by the moles of nitric acid to have the same pH value. KOH and NaOH are both strong bases, and therefore, the same amount of moles was maintained. For acids, the calculation was different since they have different acid strengths.

**Fig. 2:**
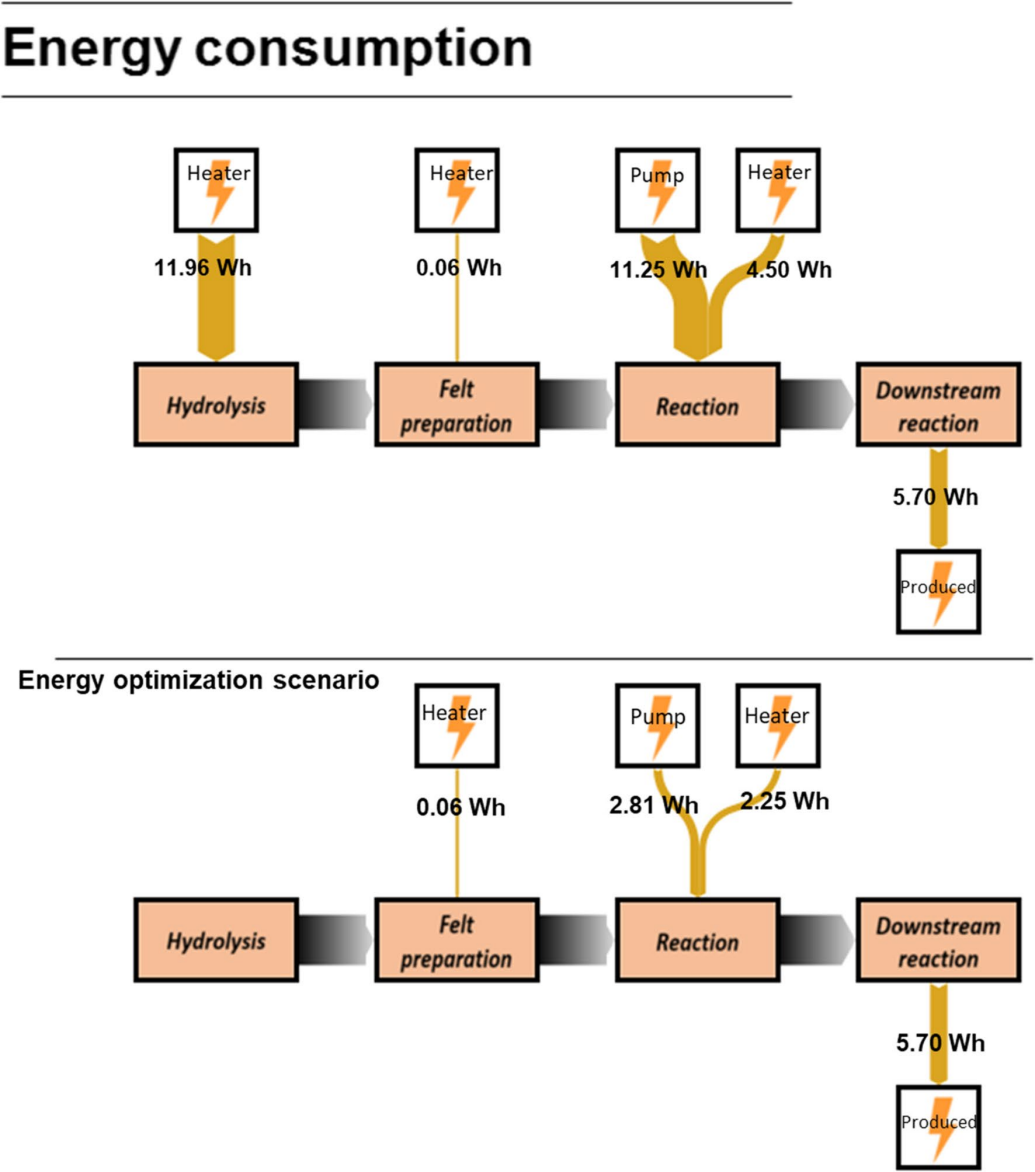
Energy consumption of the base scenario (**a**) and energy optimization scenario (**b**)

In scenario 3, the usage of a photovoltaic (PV) source of energy was evaluated. PV was selected due to its capacity to be easily incorporated on the roof of the BFC users (food companies) and its importance in the Italian market. According to the last census (Gestore Servizi Energetici 2023), the operating PV plants exceed 1.5 million (+ 23% compared to the end of 2022), for a total power of approximately 28.6 GW (+ 14%). Finally, scenario 4 represents the scaled BFC up to the industrial level, with a minimization of the energy requirements. One significant source of energy consumption comes from the use of a hot plate or stove during hydrolysis, an exothermic reaction (Setti and Maggiore 2022), as shown in Fig. 2a). The need for additional heating is avoided by harnessing the heat energy generated during this reaction (Setti and Maggiore 2022).

Another energy-intensive element was the peristaltic pump, initially oversized and not properly accounted for in terms of energy expenses. To simulate a larger-scale scenario, the pump’s four-way functionality was considered. Instead of calculating energy consumption solely from its maximum power, the *P*_max_ was divided by the number of ways (*P*_max_/4), creating a more accurate representation of its usage in a scaled-up setting. The amount of energy generated and required for the process in the two scenarios (basic and optimized) is depicted in Fig. 2 (Tables S4–5).

Both the traditional and optimized scenarios were simulated assuming the usage of electricity from the Italian energy mix (e.g. electricity, medium voltage {IT}| electricity voltage transformation from high to medium voltage | APOS).

### Software, database and methods

The LCA analysis was conducted using the SimaPro software (v.9.5) (PRè 2023). Ecoinvent (v.3.9.1) (Wernet et al. 2016) was used as the main source of background information. To fill data gap (e.g. in the case of agar), the AGRIBALYSE® database was used where the dataset was not available.

The former enables the modelling of products and systems from a life cycle perspective. The latter provides the basic information and is essential for obtaining a complete LCI. There are multiple system models in the ecoinvent database, and the allocation at the point of substitution (APOS) approach was used in this study, by selecting the market scenarios where available (to include impacts from average transportation distances). The system is assumed to be installed in Italy by assuming an average national energy mix and European deionized water production. On the other hand, worldwide processes were selected for chemical production since distributed globally. The APOS follows an attributional approach where responsibility over waste is split between producers and subsequent users who profit from the treatment processes using the valuable products produced by them. The APOS system model employs the expansion of product systems to avoid allocation within treatment systems (Wernet et al. 2016). Despite the fact the consequential approach was adopted for including our by-products in the model, we decided to adopt the APOS model to have more conservative results (i.e. potentially higher burdens). The application of a consequential approach on the entire supply chain (given by the adoption of the CONSEQ ecoinvent processes) would allow more benefits from the potential substitution of material/energy within the entire supply chain. However, there is no assurance the consequential approach is respected along the chain, since we have no direct control over it, we proceed more conservatively. The same approach was already accepted in the literature (Adeoye et al. 2023).

To assess the impacts of the streams in the inventory during the LCIA, it is necessary to select the reference analysis method. In this study, two impact methods were adopted to assess potential environmental damages by considering multiple perspectives. The first one is the Cumulative Energy Demand (CED, v.1.11) (Frischknecht et al. 2007), used as a screening indicator of the whole resources consumed by the BFC system. It is a single-issue method able to calculate “the entire demand, valued as primary energy, which arises in connection with the production, use and disposal of an economic good” (VDI - Verein Deutscher Ingenieure 1997) since it “quantifies the energy content of all different energy resources when they cross the boundary between the biosphere and the technosphere” (Frischknecht et al. 2015). The indicator separates renewable from non-renewable resources and the unit of measurement is typically expressed in energy equivalents (e.g. MJ). CED provides insight into the energy-intensive stages of a product’s life cycle and helps identify opportunities for energy efficiency improvements (Prè Sustainability 2020).

The second method used was ReCiPe 2016 (v1.08) (Huijbregts et al. 2017). It is a multiple issue tool that addresses 18 different impact categories at the intermediate or midpoint level (problem-oriented), global warming potential (GW), stratospheric ozone depletion (SOD), ionizing radiation (IR), ozone formation-human health (OF HH), fine particulate matter formation (FPMF), ozone formation-terrestrial ecosystems (OF TE), terrestrial acidification (TA), freshwater eutrophication (FWEu), marine eutrophication (Meu) terrestrial ecotoxicity (TEu), freshwater ecotoxicity (FWEc), marine ecotoxicity (MEc), human carcinogenic toxicity (HCT), human non-carcinogenic toxicity (HNCT), land use (LU), mineral (MRS) and fossil resource scarcity (FRS), and water consumption (WC). Those could be also grouped into three endpoint damage categories according to the main receptors (human health, ecosystem, and resource consumption). In addition, normalization and weighting are included in this method, allowing results to also be expressed in terms of cumulative single score (point, Pt).

## Results and discussion

The results of this study are divided into three parts, (i) the BFC reference scenario was investigated to assess the cell set-up (FU = 1p) and its usage (FU = 9.08 g treated biomass), by also evaluating the different reference service life of the apparatus; (ii) the optimized technology (BFC optimized) was addressed, aimed to study how the process could be improved for future upscaling; and (iii) the comparison with traditional scenarios for the waste biomass treatment was performed, by selecting currently established technologies available on European market.

### BFC base scenario

**Fig. 3:**
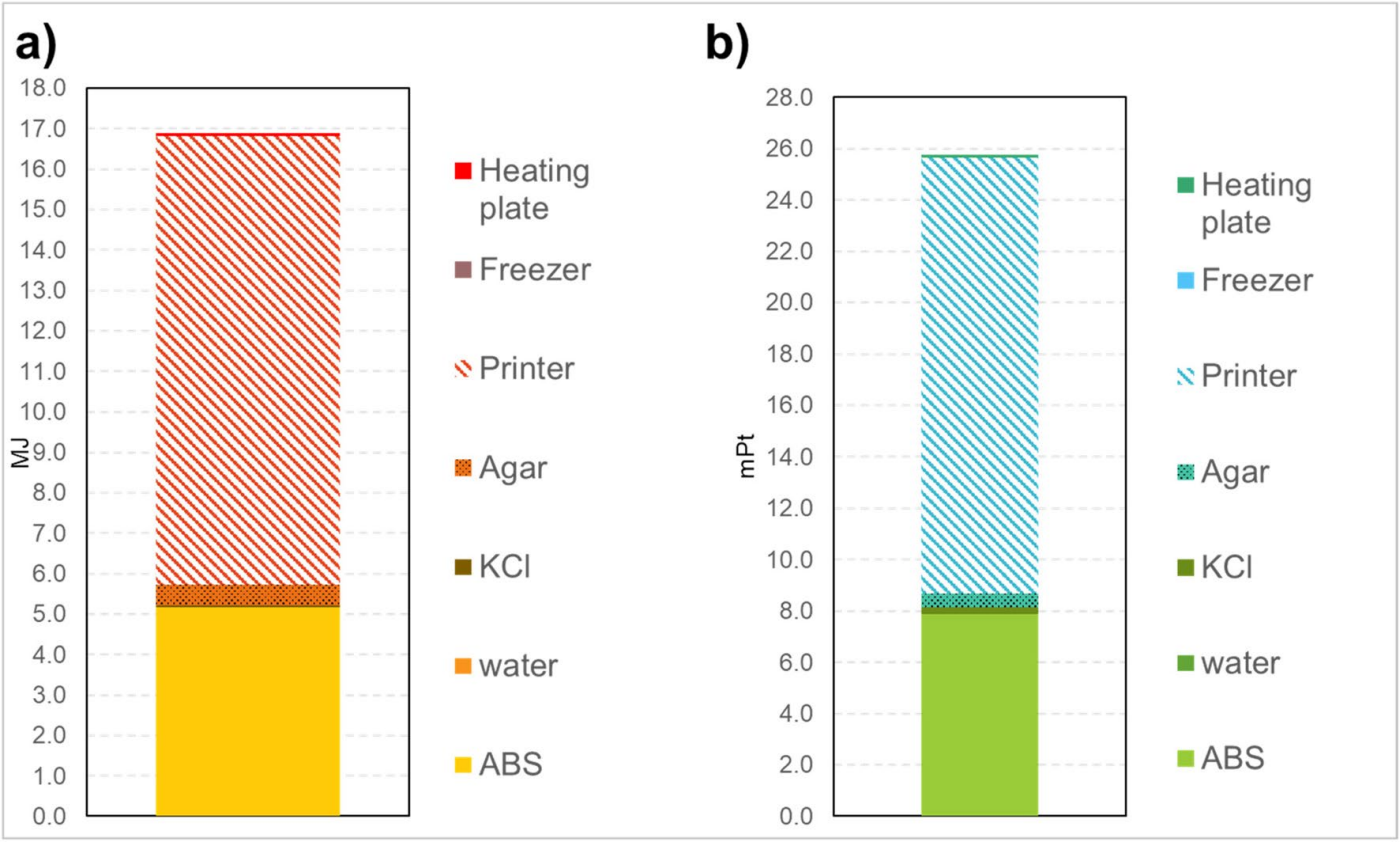
LCIA of BFC set-up (FU = 1p) in terms of **a** single-issue (Cumulative Energy Demand v1.11) and **b** multiple-issue (ReCiPe 2016 Endpoint (H) v1.08/World (2010) H/A, cumulative single score) methods

The LCIA results of the BFC set-up, by considering the FU of 1p, are shown below in Fig. 3 (Tables S6–7). The analysis depicts that the largest impacts are those related to the use of the 3D printer (66% for both methods) and those related to ABS (31% for both methods), the main constituent of the cell. Full data are given in the SI (supplementary information).

In terms of mass, the ABS accounts for 45% of the constituents, and the same percentage is related to water, which, however, has a much smaller impact for the same amount used. In fact, comparing them by the same mass, their impact varies by 4 units of magnitude. In the case of energy, the printing phase (7 h) accounts for 99.3% of the whole energy consumption.

Most of the impacts related to energy consumption come from coal energy; in fact, while it accounts for 11% of the energy mix, it accounts for 34% of the impacts using the ReCiPe 2016 method. It decreases its contribution with the CED method, where it accounts for 16%. With this second method, the impact of natural gas (40% of the mix) increases significantly from 12% (ReCiPe 2016) to 48%.

**Fig. 4:**
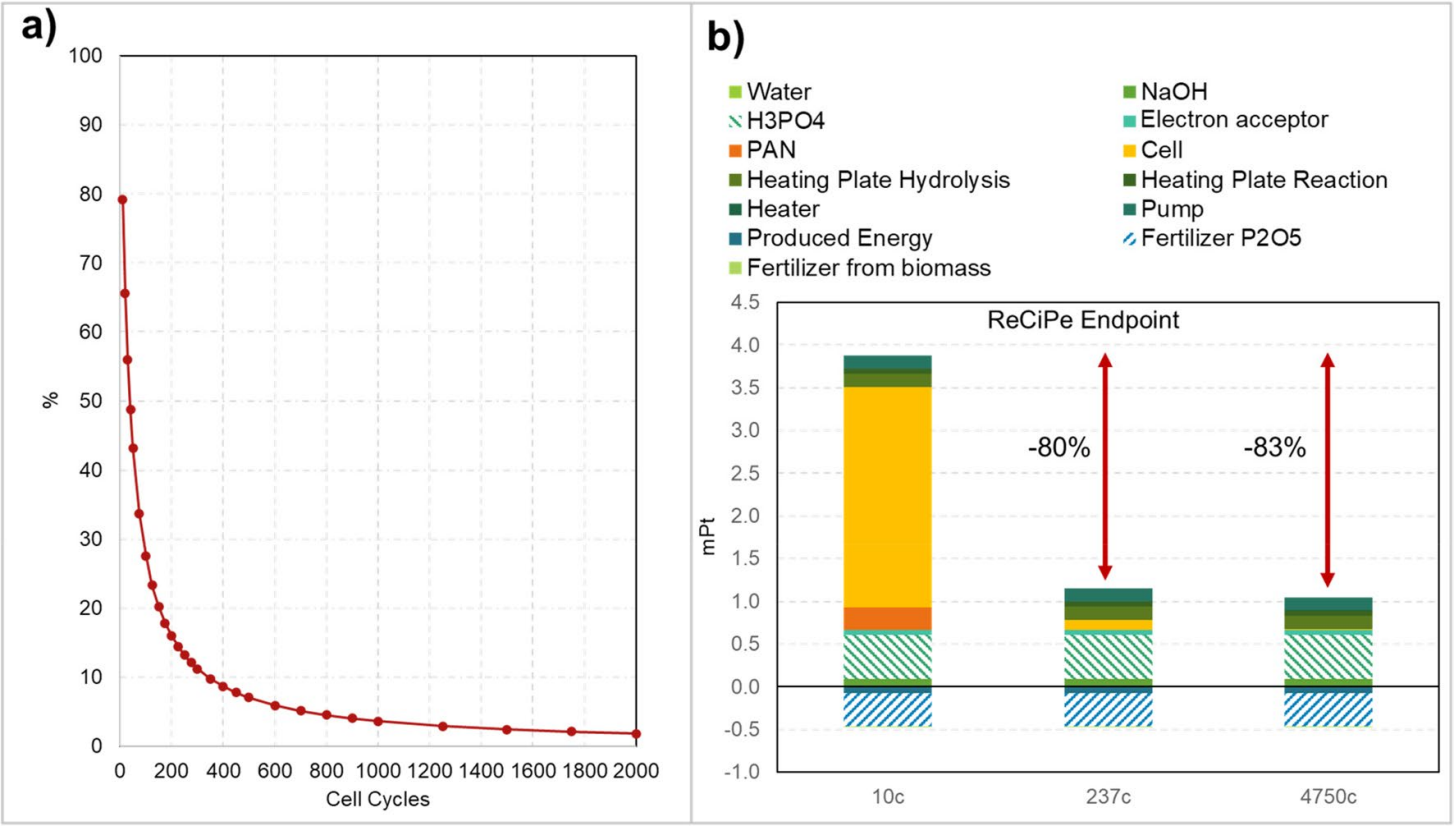
LCIA for the BFC system (FU = 9.08 g treated biomass). **a** BFC setup contribution compared to the total impact. (Cumulative Energy Demand v1.11). **b** Scenario 1 comparison in terms of cumulative single score between 10c (cycles), 237c and 4750c (ReCiPe 2016 Endpoint (H) v1.08/World (2010) H/A)

The influence of impact values linked to BFC production throughout the cell’s lifespan is depicted in Fig. 4a. This graph plots the cycles of use (*x*-axis) compared to the cumulative resources consumption in the BFC set-up (*y*-axis). CED was chosen as referring method. Notably, the graph demonstrates that around 400 cycles mark the point where the impact of manufacturing falls below 10%. Subsequently, after 1000 cycles the impacts related to the BFC assembly become nearly negligible rather than the total. Therefore, the usage of the cell for around 1000 h can be considered enough to make it competitive.

The LCIA results of scenario 1 are collected in Fig. 4b. The 10c corresponds to the baseline test carried out in the laboratory so far. Another interesting parameter related to the cell’s expectation of use is the energy break-even point, which is after how many cycles the energy spent to produce the cell is recovered. The energy consumption for producing the cell is 1270 Wh, and the energy produced is 5.4 Wh, so 237 cycles (1 cycle = 1 working h) are required to equal the energy used during manufacturing. Given the fact the energy production is one of the main goals of the technology and the consumption during production is everything than negligible, having this represents a point is of interest for the technology upscale. The calculation confirms the number of cycles required to achieve an energy gain. As reported above, each cycle is equal to 1 working hour. Therefore, considering using the cell for six cycles per day it would take only 40 days of use to amortize the energy consumption related to preparing the cell.

The latter (4750c), as written previously, is based on data found in the literature regarding the life expectancy of a common fuel cell (Hua et al. 2020). Data on impacts are given in the SI (Tables S8–9, S11–12).

The larger the cycles of use, the lower the BFC set-up contribution on the overall impact. According to the results, the cumulative single score is reduced of − 80% in the 237c configuration and of − 83% in the 4750c, with a contribution of the BFC manufacturing on the total equal to 76% in the 10c configuration that became 16% and 1% respectively for 237c and 4750c. These contributions are represented in the graph in Fig. 4a.

**Fig. 5:**
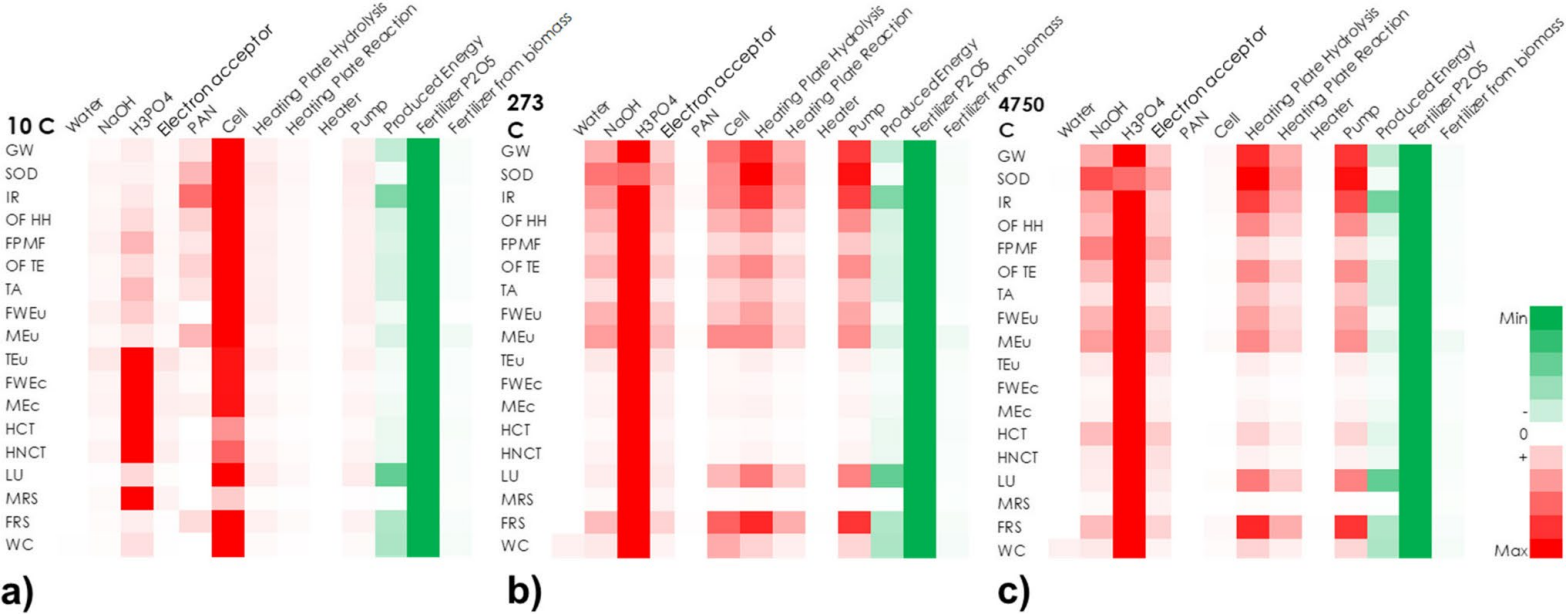
LCIA for the BFC system (FU = 9.08 g treated biomass). Scenario 1 contribution analysis in terms of midpoint: 10c (cycles), 237c and 4750c (ReCiPe 2016 Endpoint (H) v1.05/World (2010) H/A). GW, global warming; SOD, stratospheric ozone depletion; IR, ionizing radiation; OF HH, ozone formation, human health; FPMF, fine particulate matter formation; OF TE, ozone formation, terrestrial ecosystems; TA, terrestrial acidification; FWEu, freshwater eutrophication; MEu, marine eutrophication; TEu, terrestrial ecotoxicity; FWEc, freshwater ecotoxicity; MEc, marine ecotoxicity; HCT, human carcinogenic toxicity; HNCT, human non-carcinogenic toxicity; LU, land use; MRS, mineral resource scarcity; FRS, fossil resource scarcity; WC, water consumption

A further contribution analysis is reported in Fig. 5 (Tables S10, S13) in the form of color shade matrix. The graph depicts the results in terms of ReCiPe 2016 midpoint. In this way, it is easy to visualize the changing in the overall contribution of each step compared to the total impact per category.

The difference between the three scenarios is on the cell, which achieves the higher contribution in the 10c configuration. On the other hand, its impact is minimal in the case of 237c and 4750c. Here, the largest burdens are associated to the energy consumption (GW, SOD and IR) and the use of phosphoric acid (TEu). Regarding the energy consumptions they are mainly due to electricity requests for heating and pumping (11.97 and 11.25 Wh respectively). They were calculated, through approximations described in the “Life Cycle Inventory” section, using a conservative approach. While these energy consumption figures seem high, it is important to note that they could potentially decrease during the upscaling (see results of the scenario 4; the “Energy optimization (scenario 4)”). A further contribution analysis reveals the significant environmental impact associated with H_3_PO_4_ depends on its production, the wet process that implies the acid attack (H_2_SO_4_) method of phosphate minerals (Althaus et al. 2007). This process utilizes phosphate rocks, a large amount of water, and approximately 1.3 kg of H_2_SO_4_ per kg of H_3_PO_4_. The usage of sulfuric acid contributes for the 47% on the whole cumulative impact (single score). The environmental impact of using phosphoric acid is also due to its great usage compared to other reagents in the process. While water, the most abundant reagent, has a significantly lower environmental impact, the amount of H_3_PO_4_ used is three times that of sodium hydroxide (NaOH). The difference in impact is even higher by the fact that H_3_PO_4_ has a higher environmental impact per kilogram compared to NaOH when assessed using the ReCiPe 2016 method. On the other hand, using the CED method, the difference decreases (21.6 MJ/kg of H_3_PO_4_ vs 19.7 MJ/kg of NaOH). This discrepancy between the methods arises from the different weighting schemes used by each of them. The ReCiPe 2016 method assigns higher weights to environmental impacts that are considered to be more significant, such as human health and ecosystem quality. As a result, H_3_PO_4_, which has a higher impact on these areas, receives a larger weighting compared to NaOH. In the case of the base, most of the impacts come from the electricity used for the conversion of sodium chloride through electrolysis (Kurt and Bittner 2006). In fact, analyzing the impacts using the ReCiPe 2016 method shows that 12% of them are related to NaCl while almost all of the rest of the fraction is related to energy consumption.

Figure 5 illustrates the findings of the contribution analysis, also known as hotspot analysis at midpoint level by representing 18 impact categories. The figure represents the transitioning from the laboratory-scale scenario (10 cycles of use = 10c; Fig. 5a) to the upscaled system (4750 cycles of use = 4750c; Fig. 5c). As depicted in the figure, in the laboratory-scale scenario (Fig. 5a), the infrastructure (cell manufacturing) makes the greatest contribution (dark red) across most of the impact categories considered, followed by H_3_PO_4_. However, as the number of cycles (i.e. cell service life) increases, the environmental contribution of the infrastructure notably decreases (273c; Fig. 5b) to the point of being entirely negligible at 4750c usage (Fig. 5c). In the latter configuration, the H_3_PO_4_ represents the environmental hotspot.

Figure 5 also shows the largest contribution in terms of avoided impacts comes from the H_3_PO_4_ recovery and not from energy produced or nutrient recovery in biomass. Thus, the 4750c configuration was used in the optimization steps described below.

### BFC optimization

This chapter presents scenarios formulated to minimize environmental impacts. Taking an ecodesign approach, the focus was not solely on observing the current state of impacts related to the technology during the laboratory stage. Rather, alternative scenarios were crafted with the objective of optimizing the BFC and reducing the impacts for a potential industrial scale-up in the future. Following the analysis of the baseline scenario, it was observed that the predominant impacts stemmed from the reagents used and the high energy consumption, the latter being notably elevated as it has not yet undergone optimization to this TRL.

#### Reagents changing (scenario 2) and uncertainty analysis

**Fig. 6:**
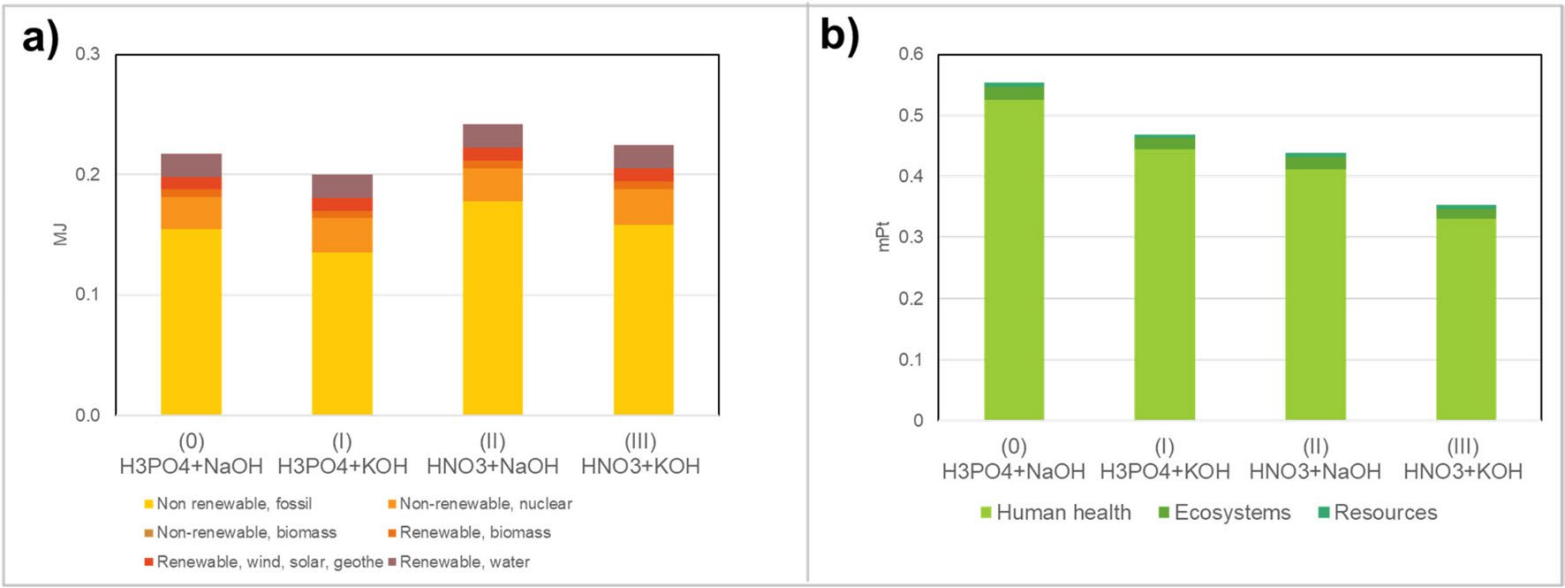
LCIA for the BFC system (FU = 9.08 g treated biomass). Scenario 2 comparison of several alternatives (0) H_3_PO_4_ + NaOH, (I) H_3_PO_4_ + KOH, (II) HNO_3_ + NaOH, (III) HNO_3_ + KOH in terms of contribution analysis in terms of **a** Cumulative Energy Demand v1.11 and **b** ReCiPe 2016 Endpoint (H) v1.08/World (2010) H/A

As written above, the phosphoric acid was selected due to its widespread use in the agri-food industry (Kurt and Bittner 2006), the target market for this process, and its ability to generate a phosphoric fertilizer as a byproduct. On the other hand, NaOH was chosen for its ease of access without any other functional reason. Thus, to minimize the process environmental impact, the acid was substituted with a stronger one: HNO_3_, which necessitates a smaller quantity. On the other hand, the sodium hydroxide was replaced with KOH to incorporate potassium into the nutrient output. The modification was implemented while preserving a constant pH. Therefore , three alternatives were simulated compared to the baseline (H_3_PO_4_ + NaOH): (I) H_3_PO_4_ + KOH, (II) HNO_3_ + NaOH, (III) HNO_3_ + KOH. Results of the sensitivity analysis are collected in Fig. 6 (complete data in Tables S14 and S15). The trend depicted by Fig. 6a distinctly reveals that the alternative which uses nitric acid and NaOH (II) is the one that achieves the worst results in terms of resources consumption (CED). However, a wider analysis conducted through the ReCiPe 2016 (Fig. 6b) ranked the alternative with nitric acid and potassium hydroxide (III) as the more viable from an environmental point of view, followed by the II. The cumulative single score, in fact, ascribed more impacts to the alternatives with H_3_PO_4_ and NaOH due to the negative effects on human health of these reagents, related to the sulfuric acid production process and the non-enrichment fertilizer output.

Concerning acid substitution, a key point is the sharp decrease in the mass of reagent needed with a consequent mitigation of the impacts, by about two times for the resource consumption and three times in the case of the multiple issue method. In the case of the base, on the other hand, there is an increase in the amount of reagent used by 40%, and for the same mass, KOH has about twice as many impacts as NaOH, but the recovery of potassium in the fertilizer output leads to a significant advantage.

Although alternative reagents are available, they still have a non-negligible environmental impact. Nitric acid is primarily produced through the catalytic Ostwald process (Thiemann et al. 2000), which involves two main steps: the oxidation of ammonia with atmospheric oxygen to produce nitrogen monoxide and the subsequent absorption of nitrogen oxides to form nitric acid. These plants typically operate at medium to high pressure (300–900 kPa). The high energetic requirements together with the usage of NH_3_ are the steps which contribute mostly.

Potassium hydroxide also is produced through electrolysis of the chloride salt, step which requires the higher amount of energy in its supply chain.

Since the alternative III seems to be most promising compared to the baseline, an uncertainty analysis was carried out. The Monte Carlo simulation was applied to both alternatives, by repeating the comparison for 1000 runs by using the two LCIA methods. The results of the analysis with the ReCiPe 2016 method cumulative single score (Figs. S1**–**3) indicate that in 94% of cases, the alternative III is less impactful than the baseline (0). The analysis of the three endpoint categories has confirmed that for the human health and ecosystem, alternative III is significantly better, with impacts lower than the baseline in 94% and 83% of cases respectively. On the contrary, there are no significant differences for the category resources consumption, where the usage of HNO_3_ and KOH seems achieve greater impacts with a frequency of 56%. This means that the alternative III would bring an environmental benefit compared to the combination of H_3_PO_4_ and NaOH. Therefore, further tests on lab scale are strongly recommended to confirm the feasibility and proceed with the upscaling.

#### Energy source substitution (scenario 3)

The energy consumption impacts are significant. According to the ReCiPe 2016 method, they account for 35% of the total impacts considering the single score. The major impacts are due to the energy consumption during the hydrolysis stage (15%) and pump usage (14%). The remaining 6% is attributed to the heating during the reaction. The CED method reveals a more significant contribution from the hydrolysis step (26%), reaction (10%), and pump use (25%), accounting for over half (61%) of the overall environmental impact on resource consumption.

Therefore, a further scenario was created by assuming an integration and substitution of the national electricity mix with that from PV technology. Two alternatives were created: one utilizing 50% solar energy alongside the traditional Italian energy mix (50%PV), and another relying entirely on 100% solar energy (100%PV). The total energy consumption, calculated at 27.8 Wh, was divided between these two energy sources. Results, collected in SI (Figs. S4–5 and Tables S16–17), show that the impacts can decrease dramatically in this way. A decrease between 22–44% for the CED and 23–46% for the ReCiPe 2016 may be achievable. This is due to the minimal environmental footprint of solar energy compared to the Italian energy mix (around − 39% of resources used).

#### Energy optimization (scenario 4)

An optimized alternative was simulated (En.Opt.) by modelling the LCI according to what reported in the “Life Cycle Inventory” section. Results of this further sensitivity analysis are collected in SI (Fig. S6). The recovery of the heat from the hydrolysis reaction and the optimization of the pump consumption may have some benefits on the whole BFC system. In the case of CED, a reduction of − 98% is achieved (Fig. S6a); on the contrary, − 58% are obtained for the ReCiPe 2016 (Fig. S6b). This disparity was expected as the first is a single-issue method aimed at stressing the resources consumption, whereas the second considers a broader spectrum of environmental factors to give a more comprehensive understanding in the case of ReCiPe 2016. Comparing the impacts, neglecting the negative impacts of avoided products, before optimization, 61% of the CED method came from energy consumption, but post-optimization only 10%. Thus, the decrease in energy consumption can lead to a significant reduction in the environmental impacts of this technology.

### Comparison with traditional biomass treatment scenarios

To examine the competitiveness of the technology under investigation, a comparison with some traditional biomass treatment techniques was carried out. Among the most used methods, anaerobic digestion (AD) and composting (COM) were selected, since they are more relevant for the Italian scenario. Both techniques were simulated using the ecoinvent default processes *Biowaste {RoW}| treatment of biowaste by anaerobic digestion | APOS, U* and *Biowaste {RoW}| treatment of biowaste, industrial composting | APOS, U*. The European process (RER) was not available in the ecoinvent database.

Since both alternatives refer to a wet mass with 40% dry matter content, to make the comparison reasonable, 22.70 g of biomass treated was considered as FU in the case of AD and COM.

As previously highlighted, one advantage of this technology is to be installed in situ. Therefore, the effect of transportation was investigated by including three distances on the AD and COM scenarios (10 km, 50 km, and 100 km) covered by truck (*Transport, freight, lorry, unspecified {RER}| transport, freight, lorry, all sizes, EURO4 to generic market for | APOS, U*).

**Fig. 7:**
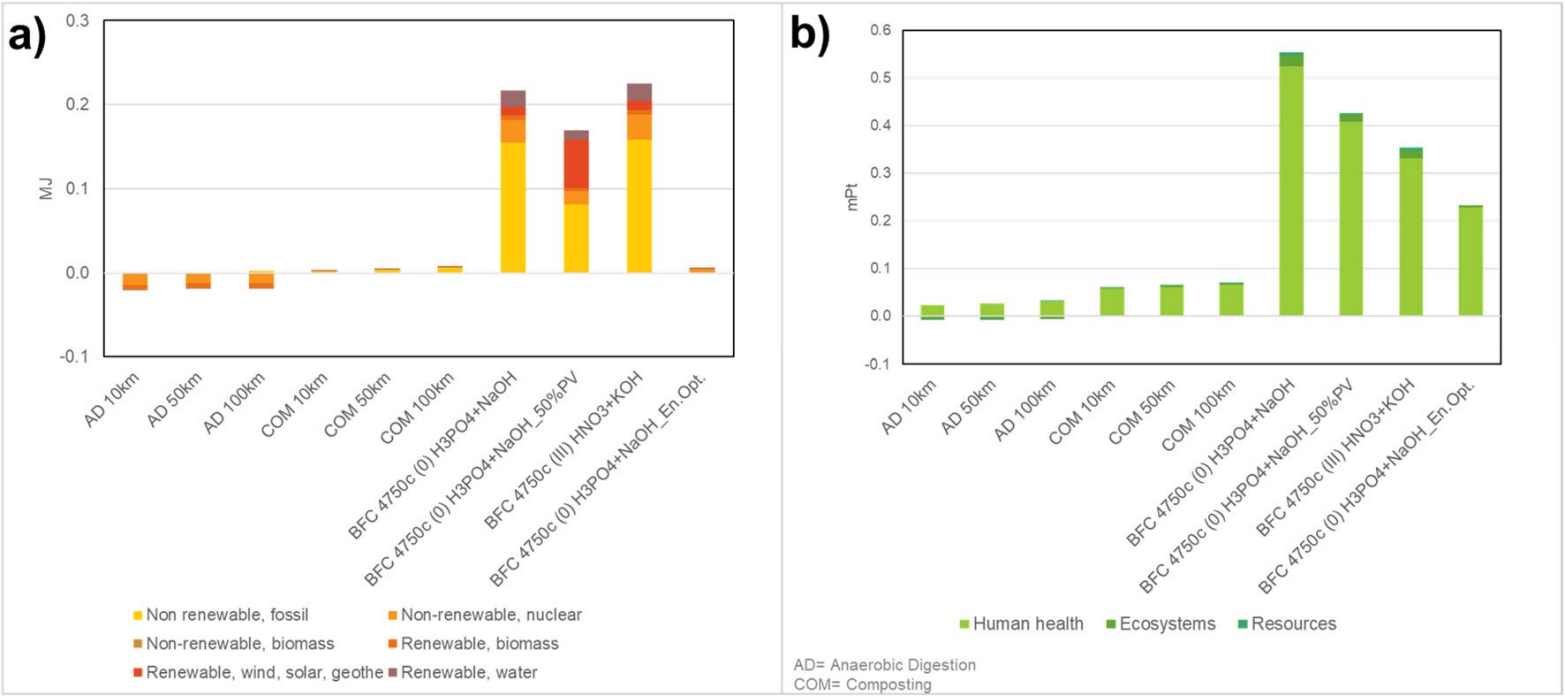
LCIA for the BFC system (FU = 9.08 g treated biomass), comparison between the BFCs and the traditional alternatives for the treatment of biowaste in terms of **a** Cumulative Energy Demand v1.11 and **b** ReCiPe 2016 Endpoint (H) v1.08/World (2010) H/A

As shown in Fig. 7a (Tables S18–19), the optimized alternative (BFC 4750c (0) H_3_PO_4_ + NaOH_En.Opt.) compared to the three non-optimized (BFC 4750c (0) H_3_PO_4_ + NaOH, BFC 4750c (0) H_3_PO_4_ + NaOH_50%PV and BFC 4750c (III) HNO_3_ + KOH) seems to compete with COM in terms of total resources consumption. Expanding the assessment to the multiple-issue method (Fig. 7b), the BFC 4750c (0) H_3_PO_4_ + NaOH_En.Opt. alternative still remains the more competitive among the BFCs, even if it is 2.7 times more impacting compared to the COM 100 km. In both cases, the AD results the best. Transportation impacts were evident in traditional scenarios, with a higher contribution in the case of CED (Fig. 7a). This suggests that the benefit of bypassing transportation primarily affects the resources exploitation, in addition to the logistical and management aspects. These results are quite expected. Traditional processes, already optimized and widespread within the industry, displayed considerably lower impacts compared to the BFCs. The latter is a new technology, which still needs further optimization. However, the comparison with the energy-optimized scenario holds promise despite the limitations previously outlined.

## Conclusion

The study presents an assessment of the potential environmental impacts related to an innovative BFC technology for treating biomass residues to co-produce energy and fertilizers. An *early-stage* LCA analysis was proposed since the BFC is developed at laboratory scale. The assessment was performed by computing the whole resources consumption and the 18 impact categories of the ReCiPe 2016 method. Several sensitivity analyses were run to address how the model could be affected by different reference service life for the BFC, the usage of alternative reagents, an increased percentage of renewables, and an optimized energy-related apparatus which simulates the BFC upscaling. Then, a comparison with more traditional processes for the biowaste treatment was performed, by selecting anaerobic digestion and composting. The results achieved for the BFC are promising and can be environmentally competitive as long as energy consumption is minimized and nutrients used among the reagents are recovered. Impacts related to reagents can be reduced by using alternatives, and the energy consumption can be mitigated by recovering the heat of reaction and using a photovoltaic system to integrate the requirements. However, the study is affected by some limitations, as summed up below. First is the scale of the process; the BFC was only tested at a laboratory scale with the use of glucose as model molecule. However, further tests are scheduled to check its behaviour on a real matrix (e.g. fruit biowaste). A production on a larger scale is also expected. Another limitation is related to the data used to complete the model. Primary data regarding the composition and the system efficiency were used to complete the LCI. However, some assumptions have been necessary to fill the data gap both in the foreground (e.g. energy and nutrients composition) and background. Secondary data were used for simulating the substitution of the starting reagents (H_3_PO_4_ + NaOH) with the alternative ones (HNO_3_ + KOH), at constant pH. Further laboratory experiments will be conducted to confirm the process successful operation with these adjustments. In this case, the *ex ante* LCA has been used as screening tool to verify the environmental sustainability of the hypothesis. Another important limitation of this study is related to outflows after the BFC treatment. It was assumed that 100% of the nutrients can be recovered, and no energy consumption was considered for the purification steps. Laboratory tests are required to estimate the exact efficiency of recovery and which are the requested. This will play a pivotal role on the scale up of the technology, given the contribution of the phosphoric acid use on the total.

Finally, comparing new technologies with mature and largely optimized technologies is relevant, but several considerations must be kept in mind to avoid misleading results. Numerous research initiatives solely furnish lab-scale data, often leading to inflated impacts when compared to commercially available materials. Optimized processes and scaling effects in commercial applications contribute to enhanced material and energy efficiencies. Consequently, the comparison may not accurately portray the potential of the developed material or process. Implementing a scale-up for comparison with competing technologies at the commercial scale can substantially enhance the environmental assessment (Piccinno et al. 2016).

## Supplementary Information

Below is the link to the electronic supplementary material.

Supplementary file1 (DOCX 852 KB)

Supplementary file2 (XLSX 12 KB)

## Data Availability

The authors declare that the data supporting the findings of this study are available within the paper and its Supplementary Information files. Should any raw data files be needed in another format they are available from the corresponding author upon reasonable request.
