# Supplementary material for: Energy from waste biomass: an LCA study on a biofuel cell at early design stage
Source: Environ Sci Pollut Res Int. 2024 Jun 26;33(22):11110–25. doi: 10.1007/s11356-024-34068-1 (PMC13415293; doi:10.1007/s11356-024-34068-1)
Supplement: Supplementary file 1 — Supplementary file1 (DOCX 852 KB) [file 11356_2024_34068_MOESM1_ESM.docx]

Supplementary Information_1 (SI_1)

# Energy from waste biomass: an LCA study on a bio-fuel cell at early design stage

**Eleonora Rossi^a,b^, Daniele Cespi^a,b,*^, Irene Maggiore^a^, Leonardo Setti^a,b^** , **Fabrizio Passarini^a,b^**

*^a^- Industrial Chemistry Deparment “Toso Montanari”, Alma Mater - Università di Bologna, Via Piero Gobetti, 85, 40136 Bologna (BO), Italy*

*^b^- Interdepartmental Center for Industrial Research Renewable Sources, Environment, Sea and Energy, University of Bologna, via Angherà 22, 47922 Rimini (RN), Italy*

Corresponding author: [daniele.cespi2@unibo.it](mailto:daniele.cespi2@unibo.it)

## Life Cycle Inventory

## LCI Cell manufacturing

**Table S 1**: BFC set-up, material inventory.

| Material | Quantity (g) | Process | Database |
| --- | --- | --- | --- |
| ABS (Acrylonitrile butadiene styrene) | 50 | Acrylonitrile-butadiene-styrene copolymer {RER}\| acrylonitrile-butadiene-styrene copolymer production \| APOS, U | ecoinvent 3 |
| H_2_O | 50 | Water, deionised {Europe without Switzerland} \| market for water, deionised \| APOS, U | ecoinvent 3 |
| KCl | 10 | Potassium chloride, industrial grade {GLO}\| market for potassium chloride, industrial grade \| APOS, U | ecoinvent 3 |
| Agar | 1.35 | Seaweed, agar, raw, processed in FR \| Ambient (long) \| LDPE \| No preparation \| at consumer/FR [Ciqual code: 11084] | AGRIBALYSE |

**Table S 2**: LCI energy of BFC set-up (FU = 1p).

| Step | Quantity (Wh) | Process | Database |
| --- | --- | --- | --- |
| Printer | 1270 | Electricity, medium voltage {IT}\| electricity voltage transformation from high to medium voltage \| APOS, U | ecoinvent 3 |
| Freezer | 0.0045 | Electricity, medium voltage {IT}\| electricity voltage transformation from high to medium voltage \| APOS, U | ecoinvent 3 |
| Heating magnetic stirrer | 8.86 | Electricity, medium voltage {IT}\| electricity voltage transformation from high to medium voltage \| APOS, U | ecoinvent 3 |

## LCI Cell Use

**Table S 3**: LCI material of BFC system (FU = 9.08g treated biomass).

| Material | Quantity (g) | Process | Database |
| --- | --- | --- | --- |
| INPUT | | | |
| H_2_O | 50.00 | Water, deionised {RoW}\| market for water, deionised \| APOS, U | ecoinvent 3 |
| NaOH | xx^*^ | Sodium hydroxide, without water, in 50% solution state {GLO}\| market for \| APOS, U | ecoinvent 3 |
| PAN | xx^*^ | Polyacrylonitrile fibres (PAN), from acrylonitrile and methacrylate, prod. mix, PAN w/o additives EU-27 S | ecoinvent 3 |
| Electron acceptor | xx^*^ | xx^*^ | xx^*^ |
| H_3_PO_4_ | xx^*^ | Phosphoric acid, industrial grade, without water, in 85% solution state {RoW}\| purification of wet-process phosphoric acid to industrial grade, product in 85% solution state \| APOS, U | ecoinvent 3 |
| AVOIDED PRODUCT | | | |
| P_2_O_5_ fertilizer (from H_3_PO_4_) | 3.40 | Organic phosphorus fertiliser, as P_2_O_5_ {GLO}\| inorganic phosphorus, as P_2_O_5_ to generic market for organic phosphorus, as P_2_O_5_ \| APOS, U | ecoinvent 3 |
| N fertilizer | 0.09 | Organic nitrogen fertiliser, as N {GLO}\| inorganic nitrogen fertiliser, as N to generic market for organic nitrogen fertiliser, as N \| APOS, U | ecoinvent 3 |
| P_2_O_5_ fertilizer | 0.003 | Organic phosphorus fertiliser, as P_2_O_5_ {GLO}\| inorganic phosphorus, as P_2_O_5_ to generic market for organic phosphorus, as P_2_O_5_ \| APOS, U | ecoinvent 3 |
| K_2_O fertilizer | 0.03 | Organic potassium fertiliser, as K_2_O {GLO}\| inorganic potassium fertiliser, as K_2_O to generic market for organic potassium fertiliser, as K_2_O \| APOS, U | ecoinvent 3 |

*: these data cannot be disclosed as they are present in the patent-pending Setti L, Maggiore I (2022) "Abiotic biofuel cell": European patent pending PCT/EP2023/083563 by ALMA MATER STUDIORUM - UNIVERSITY OF BOLOGNA

**Table S 4**: LCI energy of the BFC system (FU = 9.08g treated biomass) .

| Step | Quantity (Wh) | Process | Database |
| --- | --- | --- | --- |
| INPUT | | | |
| Heating magnetic stirrer (hydrolysis) | 11.97 | Electricity, medium voltage {IT}\| electricity voltage transformation from high to medium voltage \| APOS, U | ecoinvent 3 |
| Heater | 0.06 | Electricity, medium voltage {IT}\| electricity voltage transformation from high to medium voltage \| APOS, U | ecoinvent 3 |
| Heating magnetic stirrer (reaction) | 4.50 | Electricity, medium voltage {IT}\| electricity voltage transformation from high to medium voltage \| APOS, U | ecoinvent 3 |
| Pump | 11.25 | Electricity, medium voltage {IT}\| electricity voltage transformation from high to medium voltage \| APOS, U | ecoinvent 3 |
| AVOIDED PRODUCT | | | |
| Produced energy | 5.40 | Electricity, medium voltage {IT}\| electricity voltage transformation from high to medium voltage \| APOS, U | ecoinvent 3 |
|  |  |  |  |

## LCI Energy Optimization

**Table S 5**: Energy LCI of the baseline (Scenario 1) and the energy optimized configuration (Scenario 4)

| Input | Scenario 1 (baseline) amount (Wh) | Scenario 4 energy optimized  amount (Wh) |
| --- | --- | --- |
| Heating Magnetic Stirrer (Hydrolysis) | 11.96 | 0.00 |
| Heater | 0.06 | 0.00 |
| Heating Magnetic Stirrer (Reaction) | 4.50 | 0.00 |
| Pump | 11.25 | 2.81 |

## Life Cycle Impact Assessment

## BFC Set-up

**Table S 6**: LCIA for the BFC set-up (FU = 1p), Cumulative Energy Demand V1.11.

| **Impact category** | **Unit** | **Total** | **Acrylonitrile-butadiene-styrene copolymer, ABS, at plant/RER U** | **water, deionised {Europe without Switzerland} \| market for water, deionised \| APOS, U** | **potassium chloride, industrial grade {GLO}\| market for potassium chloride, industrial grade \| APOS, U** | **Seaweed, agar, raw, processed in FR \| Ambient (long) \| LDPE \| No preparation \| at consumer/FR** | **_ Printer _ Electricity, medium voltage {IT}\| electricity voltage transformation from high to medium voltage \| APOS, U** | **_Freezer _ Electricity, medium voltage {IT}\| electricity voltage transformation from high to medium voltage \| APOS, U** | **_ Heating_plate _ Electricity, medium voltage {IT}\| electricity voltage transformation from high to medium voltage \| APOS, U** |
| --- | --- | --- | --- | --- | --- | --- | --- | --- | --- |
| Total | MJ | 1.69E+01 | 5.17E+00 | 2.39E-04 | 6.13E-02 | 4.95E-01 | 1.11E+01 | 3.93E-05 | 7.74E-02 |
| Non renewable, fossil | MJ | 1.33E+01 | 4.92E+00 | 1.96E-04 | 5.14E-02 | 1.39E-01 | 8.13E+00 | 2.88E-05 | 5.66E-02 |
| Non-renewable, nuclear | MJ | 1.77E+00 | 2.24E-01 | 2.61E-05 | 5.44E-03 | 3.32E-01 | 1.20E+00 | 4.25E-06 | 8.35E-03 |
| Non-renewable, biomass | MJ | 1.09E-03 | 1.80E-07 | 1.02E-08 | 2.03E-06 | 1.00E-06 | 1.08E-03 | 3.83E-09 | 7.52E-06 |
| Renewable, biomass | MJ | 2.74E-01 | 8.89E-03 | 4.15E-06 | 8.25E-04 | 2.72E-03 | 2.59E-01 | 9.19E-07 | 1.81E-03 |
| Renewable, wind, solar, geothe | MJ | 5.61E-01 | 4.42E-04 | 3.67E-06 | 8.61E-04 | 4.47E-03 | 5.52E-01 | 1.96E-06 | 3.85E-03 |
| Renewable, water | MJ | 1.01E+00 | 1.42E-02 | 9.38E-06 | 2.76E-03 | 1.70E-02 | 9.65E-01 | 3.42E-06 | 6.72E-03 |

**Table S 7**: LCIA for the BFC set-up (FU = 1p), ReCiPe 2016 Endpoint (H) V1.08 / World (2010) H/A.

| **Damage category** | **Unit** | **Total** | **Acrylonitrile-butadiene-styrene copolymer, ABS, at plant/RER U** | **water, deionised {Europe without Switzerland}\| market for water, deionised \| APOS, U** | **potassium chloride, industrial grade {GLO}\| market for potassium chloride, industrial grade \| APOS, U** | **Seaweed, agar, raw, processed in FR \| Ambient (long) \| LDPE \| No preparation \| at consumer/FR** | **_ Printer _ Electricity, medium voltage {IT}\| electricity voltage transformation from high to medium voltage \| APOS, U** | **_Freezer _ Electricity, medium voltage {IT}\| electricity voltage transformation from high to medium voltage \| APOS, U** | **_ Heating_plate _ Electricity, medium voltage {IT}\| electricity voltage transformation from high to medium voltage \| APOS, U** |
| --- | --- | --- | --- | --- | --- | --- | --- | --- | --- |
| Total | mPt | 2.58E+01 | 7.87E+00 | 2.15E-03 | 2.82E-01 | 5.15E-01 | 1.70E+01 | 6.02E-05 | 1.18E-01 |
| Human health | mPt | 2.38E+01 | 7.20E+00 | 2.00E-03 | 2.20E-01 | 4.94E-01 | 1.57E+01 | 5.58E-05 | 1.10E-01 |
| Ecosystems | mPt | 1.31E+00 | 3.63E-01 | 1.49E-04 | 5.91E-02 | 1.44E-02 | 8.63E-01 | 3.06E-06 | 6.01E-03 |
| Resources | mPt | 6.94E-01 | 3.01E-01 | 8.53E-06 | 2.30E-03 | 7.08E-03 | 3.80E-01 | 1.35E-06 | 2.65E-03 |

## BFC base scenario

**Table S 8**: LCIA for the BFC system (FU = 9.08g treated biomass), Cumulative Energy Demand V1.11.

| **Impact category** | **Unit** | **Total** | **water, deionised {RoW}\| market for water, deionised \| APOS, U** | **Sodium hydroxide, without water, in 50% solution state {GLO}\| market for \| APOS, U** | **Phosphoric acid, industrial grade, without water, in 85% solution state {RoW}\| purification of wet-process phosphoric acid to industrial grade, product in 85% solution state \| APOS, U** | **Electron aceptor** | **Polyacrylonitrile fibres (PAN), from acrylonitrile and methacrylate, prod. mix, PAN w/o additives EU-27 S** | **_ Heating_plate _Hydolysis_ Electricity, medium voltage {IT}\| electricity voltage transformation from high to medium voltage \| APOS, U** | **_ Heating_plate _Reaction_ Electricity, medium voltage {IT}\| electricity voltage transformation from high to medium voltage \| APOS, U** | **_Heater_ Electricity, medium voltage {IT}\| electricity voltage transformation from high to medium voltage \| APOS, U** | **_Pump _ Electricity, medium voltage {IT}\| electricity voltage transformation from high to medium voltage \| APOS, U** | **_Produced _ Electricity, medium voltage {IT}\| electricity voltage transformation from high to medium voltage \| APOS, U** | **organic phosphorus fertiliser, as P2O5 {GLO}\| inorganic phosphorus, as P2O5 to generic market for organic phosphorus, as P2O5 \| APOS, U** | **fertilizer from bio-fuel cell from biomass per kg bio-genic waste** |
| --- | --- | --- | --- | --- | --- | --- | --- | --- | --- | --- | --- | --- | --- | --- |
| Non renewable, fossil | MJ | 1.69E+00 | 2.00E-04 | 2.41E-02 | 9.02E-02 | 1.57E-02 | 2.05E-01 | 7.65E-02 | 2.88E-02 | 3.71E-04 | 7.20E-02 | -3.45E-02 | -1.14E-01 | -3.09E-03 |
| Non-renewable, nuclear | MJ | 2.22E-01 | 2.06E-05 | 4.30E-03 | 4.43E-03 | 2.17E-03 | 1.85E-02 | 1.13E-02 | 4.25E-03 | 5.47E-05 | 1.06E-02 | -5.10E-03 | -4.93E-03 | -9.43E-05 |
| Non-renewable, biomass | MJ | 1.30E-04 | 1.09E-08 | 1.86E-06 | 3.23E-05 | 2.50E-06 | 0.00E+00 | 1.02E-05 | 3.83E-06 | 4.93E-08 | 9.56E-06 | -4.59E-06 | -3.43E-05 | -3.57E-07 |
| Renewable, biomass | MJ | 3.34E-02 | 4.75E-06 | 7.07E-04 | 2.66E-03 | 8.31E-04 | 5.34E-07 | 2.44E-03 | 9.19E-04 | 1.18E-05 | 2.30E-03 | -1.10E-03 | -2.73E-03 | -5.64E-05 |
| Renewable, wind, solar, geothe | MJ | 6.64E-02 | 3.06E-06 | 5.88E-04 | 6.17E-04 | 2.90E-04 | 9.22E-05 | 5.20E-03 | 1.96E-03 | 2.52E-05 | 4.89E-03 | -2.35E-03 | -9.89E-04 | -1.65E-05 |
| Renewable, water | MJ | 1.22E-01 | 1.06E-05 | 1.81E-03 | 3.47E-03 | 1.14E-03 | 2.28E-03 | 9.08E-03 | 3.42E-03 | 4.40E-05 | 8.54E-03 | -4.10E-03 | -4.14E-03 | -6.92E-05 |

**Table 9**: LCIA for the BFC system (FU = 9.08g treated biomass), ReCiPe 2016 Endpoint (H) V1.08 / World (2010) H/A.

| **Damage category** | **Unit** | **Total** | **water, deionised {RoW}\| market for water, deionised \| APOS, U** | **Sodium hydroxide, without water, in 50% solution state {GLO}\| market for \| APOS, U** | **Phosphoric acid, industrial grade, without water, in 85% solution state {RoW}\| purification of wet-process phosphoric acid to industrial grade, product in 85% solution state \| APOS, U** | **Electron Acceptor** | **Polyacrylonitrile fibres (PAN), from acrylonitrile and methacrylate, prod. mix, PAN w/o additives EU-27 S** | **_ Heating_plate _Hydolysis_ Electricity, medium voltage {IT}\| electricity voltage transformation from high to medium voltage \| APOS, U** | **_ Heating_plate _Reaction_ Electricity, medium voltage {IT}\| electricity voltage transformation from high to medium voltage \| APOS, U** | **_Heater_ Electricity, medium voltage {IT}\| electricity voltage transformation from high to medium voltage \| APOS, U** | **_Pump _ Electricity, medium voltage {IT}\| electricity voltage transformation from high to medium voltage \| APOS, U** | **_Produced _ Electricity, medium voltage {IT}\| electricity voltage transformation from high to medium voltage \| APOS, U** | **organic phosphorus fertiliser, as P2O5 {GLO}\| inorganic phosphorus, as P2O5 to generic market for organic phosphorus, as P2O5 \| APOS, U** | **fertilizer from bio-fuel cell from biomass per kg bio-genic waste** |
| --- | --- | --- | --- | --- | --- | --- | --- | --- | --- | --- | --- | --- | --- | --- |
| Total | mPt | 3.40E+00 | 2.18E-03 | 8.98E-02 | 5.11E-01 | 6.40E-02 | 2.58E-01 | 1.60E-01 | 6.02E-02 | 7.76E-04 | 1.51E-01 | -7.22E-02 | -3.96E-01 | -8.70E-03 |
| Human health | mPt | 3.15E+00 | 2.02E-03 | 8.62E-02 | 4.90E-01 | 6.14E-02 | 2.37E-01 | 1.48E-01 | 5.58E-02 | 7.19E-04 | 1.40E-01 | -6.70E-02 | -3.76E-01 | -8.22E-03 |
| Ecosystems | mPt | 1.63E-01 | 1.49E-04 | 2.78E-03 | 1.65E-02 | 2.07E-03 | 9.74E-03 | 8.13E-03 | 3.06E-03 | 3.94E-05 | 7.64E-03 | -3.67E-03 | -1.41E-02 | -3.27E-04 |
| Resources | mPt | 8.76E-02 | 8.55E-06 | 7.71E-04 | 5.00E-03 | 5.53E-04 | 1.11E-02 | 3.58E-03 | 1.35E-03 | 1.74E-05 | 3.37E-03 | -1.62E-03 | -5.71E-03 | -1.47E-04 |

**Table S 10**: LCIA for the BFC system (FU = 9.08g treated biomass), ReCiPe 2016 Midpoint (H) V1.08 / World (2010) H/A.

| **Impact category** | **Unit** | **Total** | **water, deionised {RoW}\| market for water, deionised \| APOS, U** | **Sodium hydroxide, without water, in 50% solution state {GLO}\| market for \| APOS, U** | **Phosphoric acid, industrial grade, without water, in 85% solution state {RoW}\| purification of wet-process phosphoric acid to industrial grade, product in 85% solution state \| APOS, U** | **Electron Acceptor** | **Polyacrylonitrile fibres (PAN), from acrylonitrile and methacrylate, prod. mix, PAN w/o additives EU-27 S** | **_ Heating_plate _Hydolysis_ Electricity, medium voltage {IT}\| electricity voltage transformation from high to medium voltage \| APOS, U** | **_ Heating_plate _Reaction_ Electricity, medium voltage {IT}\| electricity voltage transformation from high to medium voltage \| APOS, U** | **_Heater_ Electricity, medium voltage {IT}\| electricity voltage transformation from high to medium voltage \| APOS, U** | **_Pump _ Electricity, medium voltage {IT}\| electricity voltage transformation from high to medium voltage \| APOS, U** | **_Produced _ Electricity, medium voltage {IT}\| electricity voltage transformation from high to medium voltage \| APOS, U** | **organic phosphorus fertiliser, as P2O5 {GLO}\| inorganic phosphorus, as P2O5 to generic market for organic phosphorus, as P2O5 \| APOS, U** | **fertilizer from bio-fuel cell from biomass per kg bio-genic waste** |
| --- | --- | --- | --- | --- | --- | --- | --- | --- | --- | --- | --- | --- | --- | --- |
| Global warming | kg CO_2_ _eq_ | 1.03E-01 | 1.63E-05 | 2.10E-03 | 6.50E-03 | 1.37E-03 | 9.67E-03 | 5.39E-03 | 2.03E-03 | 2.61E-05 | 5.07E-03 | -2.43E-03 | -1.03E-02 | -2.60E-04 |
| Stratospheric ozone depletion | kg CFC11 _eq_ | 1.65E-08 | 1.50E-11 | 2.27E-09 | 2.51E-09 | 1.23E-09 | 1.33E-08 | 4.16E-09 | 1.57E-09 | 2.02E-11 | 3.91E-09 | -1.88E-09 | -5.52E-08 | -1.50E-09 |
| Ionizing radiation | kBq Co-60 _eq_ | 9.80E-04 | 1.05E-07 | 2.05E-05 | 5.14E-05 | 1.11E-05 | 3.11E-04 | 4.11E-05 | 1.55E-05 | 2.00E-07 | 3.87E-05 | -1.86E-05 | -3.71E-05 | -6.88E-07 |
| Ozone formation, Human health | kg NOx _eq_ | 1.86E-04 | 3.72E-08 | 5.53E-06 | 1.99E-05 | 4.02E-06 | 2.50E-05 | 9.33E-06 | 3.51E-06 | 4.52E-08 | 8.78E-06 | -4.21E-06 | -2.60E-05 | -5.33E-07 |
| Fine particulate matter formation | kg PM2.5_eq_ | 1.13E-04 | 3.89E-08 | 4.53E-06 | 2.35E-05 | 3.09E-06 | 8.22E-06 | 5.47E-06 | 2.06E-06 | 2.65E-08 | 5.14E-06 | -2.47E-06 | -1.78E-05 | -3.37E-07 |
| Ozone formation, Terrestrial ecosystems | kg NOx_eq_ | 1.91E-04 | 3.78E-08 | 5.58E-06 | 2.03E-05 | 4.07E-06 | 2.54E-05 | 9.49E-06 | 3.57E-06 | 4.60E-08 | 8.93E-06 | -4.28E-06 | -2.65E-05 | -5.45E-07 |
| Terrestrial acidification | kg SO_2eq_ | 3.26E-04 | 9.06E-08 | 7.66E-06 | 6.48E-05 | 5.43E-06 | 2.67E-05 | 1.63E-05 | 6.12E-06 | 7.89E-08 | 1.53E-05 | -7.35E-06 | -4.76E-05 | -9.97E-07 |
| Freshwater eutrophication | kg P _eq_ | 1.60E-06 | 6.50E-10 | 1.16E-07 | 3.89E-07 | 8.07E-08 | 7.89E-09 | 1.43E-07 | 5.37E-08 | 6.92E-10 | 1.34E-07 | -6.44E-08 | -1.21E-06 | -9.32E-09 |
| Marine eutrophication | kg N _eq_ | 1.64E-06 | 2.68E-10 | 4.27E-08 | 1.09E-07 | 2.91E-08 | 3.44E-07 | 5.05E-08 | 1.90E-08 | 2.45E-10 | 4.75E-08 | -2.28E-08 | -1.67E-07 | -8.83E-09 |
| Terrestrial ecotoxicity | kg 1,4-DCB | 7.11E-02 | 5.67E-05 | 3.92E-03 | 4.32E-02 | 4.62E-03 | 1.65E-03 | 2.98E-03 | 1.12E-03 | 1.45E-05 | 2.80E-03 | -1.35E-03 | -2.71E-02 | -6.08E-04 |
| Freshwater ecotoxicity | kg 1,4-DCB | 7.03E-05 | 6.48E-08 | 1.74E-06 | 4.24E-05 | 2.55E-06 | 7.15E-07 | 1.39E-06 | 5.24E-07 | 6.76E-09 | 1.31E-06 | -6.29E-07 | -1.88E-05 | -3.92E-07 |
| Marine ecotoxicity | kg 1,4-DCB | 1.81E-04 | 1.27E-07 | 4.96E-06 | 1.02E-04 | 7.05E-06 | 6.41E-06 | 5.31E-06 | 2.00E-06 | 2.58E-08 | 5.00E-06 | -2.40E-06 | -4.43E-05 | -8.41E-07 |
| Human carcinogenic toxicity | kg 1,4-DCB | 1.95E-03 | 6.21E-07 | 3.76E-05 | 1.39E-03 | 6.58E-05 | 4.60E-06 | 3.52E-05 | 1.33E-05 | 1.71E-07 | 3.31E-05 | -1.59E-05 | -1.95E-04 | -4.63E-06 |
| Human non-carcinogenic toxicity | kg 1,4-DCB | 1.86E-02 | 8.85E-06 | 5.86E-04 | 1.21E-02 | 8.16E-04 | 1.19E-04 | 5.98E-04 | 2.25E-04 | 2.90E-06 | 5.63E-04 | -2.70E-04 | -3.52E-03 | -5.93E-05 |
| Land use | m^2^a crop _eq_ | 1.72E-02 | 9.35E-07 | 1.45E-04 | 2.05E-03 | 1.91E-04 | 0.00E+00 | 1.06E-03 | 4.00E-04 | 5.16E-06 | 1.00E-03 | -4.80E-04 | -7.85E-04 | -1.41E-05 |
| Mineral resource scarcity | kg Cu _eq_ | 7.43E-05 | 1.44E-07 | 6.69E-06 | 2.86E-04 | 1.49E-05 | 7.88E-07 | 3.54E-06 | 1.33E-06 | 1.72E-08 | 3.33E-06 | -1.60E-06 | -2.95E-04 | -3.42E-06 |
| Fossil resource scarcity | kg oil _eq_ | 3.65E-02 | 4.37E-06 | 5.25E-04 | 1.97E-03 | 3.41E-04 | 4.00E-03 | 1.67E-03 | 6.29E-04 | 8.11E-06 | 1.57E-03 | -7.55E-04 | -2.49E-03 | -6.76E-05 |
| Water consumption | m^3^ | 5.49E-03 | 3.49E-05 | 5.60E-05 | 6.02E-04 | 2.89E-05 | 1.66E-07 | 1.05E-04 | 3.93E-05 | 5.07E-07 | 9.83E-05 | -4.72E-05 | -1.46E-04 | -5.33E-06 |

**Table S 11**: LCIA for the BFC system (FU = 9.08g treated biomass) Scenario 1 comparison: 10c (cycles), 237c and 4750c, Cumulative Energy Demand V1.11.

| **Impact category** | **Unit** | **10c** | **237c** | **4750c** |
| --- | --- | --- | --- | --- |
| Non renewable, fossil | MJ | 1.33E+00 | 5.61E-02 | 2.80E-03 |
| Non-renewable, nuclear | MJ | 1.77E-01 | 7.46E-03 | 3.72E-04 |
| Non-renewable, biomass | MJ | 1.09E-04 | 4.60E-06 | 2.30E-07 |
| Renewable, biomass | MJ | 2.74E-02 | 1.15E-03 | 5.76E-05 |
| Renewable, wind, solar, geothe | MJ | 5.61E-02 | 2.37E-03 | 1.18E-04 |
| Renewable, water | MJ | 1.01E-01 | 4.24E-03 | 2.12E-04 |

**Table S 12**: LCIA for the BFC system (FU = 9.08g treated biomass) Scenario 1 comparison: 10c (cycles), 237c and 4750c, ReCiPe 2016 Endpoint (H) V1.08 / World (2010) H/A.

| **Damage category** | **Unit** | **10c** | **237c** | **4750c** |
| --- | --- | --- | --- | --- |
| Total | mPt | 2.58E+00 | 1.09E-01 | 5.43E-03 |
| Human health | mPt | 2.38E+00 | 1.00E-01 | 5.01E-03 |
| Ecosystems | mPt | 1.31E-01 | 5.51E-03 | 2.75E-04 |
| Resources | mPt | 6.94E-02 | 2.93E-03 | 1.46E-04 |

**Table S 13**: LCIA for the BFC system (FU = 9.08g treated biomass) Scenario 1 comparison: 10c (cycles), 237c and 4750c, ReCiPe 2016 Midpoint (H) V1.08 / World (2010) H/A.

| **Impact category** | **Unit** | **10c** | **237c** | **4750c** |
| --- | --- | --- | --- | --- |
| Global warming | kg CO_2_ _eq_ | 8.36E-02 | 3.53E-03 | 2.08E-04 |
| Stratospheric ozone depletion | kg CFC11 _eq_ | 4.61E-08 | 1.95E-09 | 8.43E-11 |
| Ionizing radiation | kBq Co-60 _eq_ | 5.47E-04 | 2.31E-05 | 8.49E-07 |
| Ozone formation, Human health | kg NOx _eq_ | 1.40E-04 | 5.92E-06 | 2.95E-07 |
| Fine particulate matter formation | kg PM2.5_eq_ | 8.14E-05 | 3.43E-06 | 2.53E-08 |
| Ozone formation, Terrestrial ecosystems | kg NOx_eq_ | 1.45E-04 | 6.12E-06 | 3.06E-07 |
| Terrestrial acidification | kg SO_2eq_ | 2.40E-04 | 1.01E-05 | 5.05E-07 |
| Freshwater eutrophication | kg P _eq_ | 1.96E-06 | 8.26E-08 | 4.12E-09 |
| Marine eutrophication | kg N _eq_ | 1.19E-06 | 5.03E-08 | 2.51E-09 |
| Terrestrial ecotoxicity | kg 1,4-DCB | 3.98E-02 | 1.68E-03 | 3.58E-05 |
| Freshwater ecotoxicity | kg 1,4-DCB | 3.94E-05 | 1.66E-06 | 6.38E-08 |
| Marine ecotoxicity | kg 1,4-DCB | 9.53E-05 | 4.02E-06 | 4.51E-08 |
| Human carcinogenic toxicity | kg 1,4-DCB | 5.93E-04 | 2.50E-05 | 1.37E-08 |
| Human non-carcinogenic toxicity | kg 1,4-DCB | 7.46E-03 | 3.15E-04 | 6.56E-07 |
| Land use | m^2^a crop _eq_ | 1.36E-02 | 5.75E-04 | 2.87E-05 |
| Mineral resource scarcity | kg Cu _eq_ | 5.75E-05 | 2.43E-06 | 9.85E-08 |
| Fossil resource scarcity | kg oil _eq_ | 2.91E-02 | 1.23E-03 | 6.12E-05 |
| Water consumption | m^3^ | 4.73E-03 | 1.99E-04 | 9.95E-06 |

## Reagents changing (Scenario 2) and uncertainty analysis

**Table S 14**: LCIA for the BFC system (FU = 9.08g treated biomass), Scenario 2 (III) HNO3+KOH, Cumulative Energy Demand V1.11.

| **Impact category** | **Unit** | **Potassium hydroxide {GLO}\| market for \| APOS, U** | **Nitric acid, without water, in 50% solution state {RoW}\| nitric acid production, product in 50% solution state \| APOS, U** | **organic nitrogen fertiliser, as N {GLO}\| inorganic nitrogen fertiliser, as N to generic market for organic nitrogen fertiliser, as N \| APOS, U** | **organic potassium fertiliser, as K2O {GLO}\| inorganic potassium fertiliser, as K2O to generic market for organic potassium fertiliser, as K2O \| APOS, U** |
| --- | --- | --- | --- | --- | --- |
| Non renewable, fossil | MJ | 7.50E-02 | 3.53E-02 | -5.37E-02 | -7.02E-02 |
| Non-renewable, nuclear | MJ | 8.85E-03 | 7.89E-04 | -1.56E-03 | -2.63E-03 |
| Non-renewable, biomass | MJ | 4.49E-06 | 8.43E-07 | -6.02E-06 | -8.43E-06 |
| Renewable, biomass | MJ | 2.18E-03 | 7.08E-04 | -9.44E-04 | -1.51E-03 |
| Renewable, wind, solar, geothe | MJ | 1.21E-03 | 9.86E-05 | -2.76E-04 | -4.45E-04 |
| Renewable, water | MJ | 3.87E-03 | 4.07E-04 | -1.15E-03 | -1.87E-03 |

**Table S 15**: LCIA for the BFC system (FU = 9.08g treated biomass), Scenario 2 (III) HNO3+KOH, ReCiPe 2016 Endpoint (H) V1.08 / World (2010) H/A.

| **Damage category** | **Unit** | **Potassium hydroxide {GLO}\| market for \| APOS, U** | **Nitric acid, without water, in 50% solution state {RoW}\| nitric acid production, product in 50% solution state \| APOS, U** | **organic nitrogen fertiliser, as N {GLO}\| inorganic nitrogen fertiliser, as N to generic market for organic nitrogen fertiliser, as N \| APOS, U** | **organic potassium fertiliser, as K2O {GLO}\| inorganic potassium fertiliser, as K2O to generic market for organic potassium fertiliser, as K2O \| APOS, U** |
| --- | --- | --- | --- | --- | --- |
| Total | mPt | 2.39E-01 | 1.60E-01 | -1.46E-01 | -2.35E-01 |
| Human health | mPt | 2.28E-01 | 1.51E-01 | -1.38E-01 | -2.22E-01 |
| Ecosystems | mPt | 7.83E-03 | 6.79E-03 | -5.46E-03 | -8.91E-03 |
| Resources | mPt | 2.96E-03 | 1.50E-03 | -2.55E-03 | -3.36E-03 |

**Table S 15**: LCIA for the BFC system (FU = 9.08g treated biomass), Scenario 2 (III) HNO3+KOH, ReCiPe 2016 Endpoint (H) V1.08 / World (2010) H/A.

**Table S 16**: LCIA for the BFC system (FU = 9.08g treated biomass), Scenario 3 comparison of traditional Italian energy mix, 50%PV and 100%PV, Cumulative Energy Demand V1.11.

| **Impact category** | **Unit** | **50%PV** | | **100%PV** |
| --- | --- | --- | --- | --- |
|  |  | **Electricity, medium voltage {IT}\| electricity voltage transformation from high to medium voltage \| APOS, U** | **Electricity, low voltage, label-certified {CH}\| electricity production, photovoltaic, 3kWp slanted-roof installation, single-Si, panel, mounted, label-certified \| APOS, U** | **Electricity, low voltage, label-certified {CH}\| electricity production, photovoltaic, 3kWp slanted-roof installation, single-Si, panel, mounted, label-certified \| APOS, U** |
| Non renewable, fossil | MJ | 8.88E-02 | 1.55E-02 | 3.10E-02 |
| Non-renewable, nuclear | MJ | 1.31E-02 | 2.04E-03 | 4.09E-03 |
| Renewable, water | MJ | 1.05E-02 | 1.91E-03 | 3.89E-06 |
| Renewable, wind, solar, geothe | MJ | 6.03E-03 | 5.37E-02 | 1.10E-03 |
| Renewable, biomass | MJ | 2.84E-03 | 5.52E-04 | 1.07E-01 |
| Non-renewable, biomass | MJ | 1.18E-05 | 1.94E-06 | 3.81E-03 |

**Table S 17**: LCIA for the BFC system (FU = 9.08g treated biomass), Scenario 3 comparison of traditional Italian energy mix, 50%PV and 100%PV, ReCiPe 2016 Endpoint (H) V1.08 / World (2010) H/A.

| **Damage category** | **Unit** | **50%PVr** | | **100%PVr** |
| --- | --- | --- | --- | --- |
|  |  | **Electricity, medium voltage {IT}\| electricity voltage transformation from high to medium voltage \| APOS, U** | **Electricity, low voltage, label-certified {CH}\| electricity production, photovoltaic, 3kWp slanted-roof installation, single-Si, panel, mounted, label-certified \| APOS, U** | **Electricity, low voltage, label-certified {CH}\| electricity production, photovoltaic, 3kWp slanted-roof installation, single-Si, panel, mounted, label-certified \| APOS, U** |
| Total | mPt | 1.86E-01 | 5.84E-02 | 1.17E-01 |
| Human health | mPt | 1.72E-01 | 5.59E-02 | 1.12E-01 |
| Ecosystems | mPt | 9.43E-03 | 1.88E-03 | 3.77E-03 |
| Resources | mPt | 4.16E-03 | 5.87E-04 | 1.17E-03 |

## Energy Optimization

- 1. **Comparison with traditional biomass treatment scenarios**

**Table S 18**: LCIA for the BFC system (FU = 9.08g treated biomass), comparison between the BFCs and the traditional alternatives for the treatment of biowaste, Cumulative Energy Demand v1.11.

| **Impact category** | **Unit** | **AD 10km** | **AD 50km** | **AD 100km** | **COM 10km** | **COM 50km** | **COM 100km** | **BFC 4750c (0) H_3_PO_4_+NaOH** | **BFC 4750c (0) H_3_PO_4_+NaOH_50%PV** | **BFC 4750c (III) HNO_3_+KOH** | **BFC 4750c (0) H_3_PO_4_+NaOH_En.Opt.** |
| --- | --- | --- | --- | --- | --- | --- | --- | --- | --- | --- | --- |
| Non renewable, fossil | MJ | -1.83E-03 | 2.82E-05 | 2.35E-03 | 1.52E-03 | 3.42E-03 | 5.79E-03 | 1.54E-01 | 8.09E-02 | 1.58E-01 | -7.90E-04 |
| Non-renewable, nuclear | MJ | -1.22E-02 | -1.22E-02 | -1.21E-02 | 4.06E-04 | 4.31E-04 | 4.62E-04 | 2.72E-02 | 1.62E-02 | 2.96E-02 | 3.83E-03 |
| Non-renewable, biomass | MJ | -6.05E-05 | -6.05E-05 | -6.04E-05 | -3.55E-07 | -3.09E-07 | -2.50E-07 | 2.07E-05 | 1.09E-05 | 1.68E-05 | 4.41E-08 |
| Renewable, biomass | MJ | -5.79E-03 | -5.79E-03 | -5.78E-03 | 5.95E-04 | 6.01E-04 | 6.08E-04 | 5.96E-03 | 3.68E-03 | 5.99E-03 | 9.48E-04 |
| Renewable, wind, solar, geothe | MJ | -6.69E-05 | -6.22E-05 | -5.64E-05 | 6.47E-05 | 6.79E-05 | 7.19E-05 | 1.03E-02 | 5.80E-02 | 1.08E-02 | -5.14E-04 |
| Renewable, water | MJ | -2.91E-04 | -2.77E-04 | -2.60E-04 | 1.07E-04 | 1.19E-04 | 1.35E-04 | 1.93E-02 | 1.07E-02 | 2.02E-02 | 4.67E-04 |

**Table S 19**: LCIA for the BFC system (FU = 9.08g treated biomass), comparison between the BFCs and the traditional alternatives for the treatment of biowaste, ReCiPe 2016 Endpoint (H) V1.08 / World (2010) H/A.

| **Damage category** | **Unit** | **AD 10km** | **AD 50km** | **AD 100km** | **COM 10km** | **COM 50km** | **COM 100km** | **BFC 4750c (0) H_3_PO_4_+NaOH** | **BFC 4750c (0) H_3_PO_4_+NaOH_50%PV** | **BFC 4750c (III) HNO_3_+KOH** | **BFC 4750c (0) H_3_PO_4_+NaOH_En.Opt.** |
| --- | --- | --- | --- | --- | --- | --- | --- | --- | --- | --- | --- |
| Total | mPt | 1.69E-02 | 2.08E-02 | 2.56E-02 | 6.04E-02 | 6.44E-02 | 6.95E-02 | 5.54E-01 | 4.27E-01 | 3.54E-01 | 1.69E-02 |
| Human health | mPt | 2.32E-02 | 2.67E-02 | 3.12E-02 | 5.76E-02 | 6.13E-02 | 6.60E-02 | 5.25E-01 | 4.09E-01 | 3.31E-01 | 2.32E-02 |
| Ecosystems | mPt | -6.05E-03 | -5.89E-03 | -5.70E-03 | 2.70E-03 | 2.85E-03 | 3.05E-03 | 2.20E-02 | 1.45E-02 | 1.58E-02 | -6.05E-03 |
| Resources | mPt | -1.90E-04 | -6.43E-05 | 9.24E-05 | 1.38E-04 | 2.63E-04 | 4.20E-04 | 7.09E-03 | 3.53E-03 | 6.61E-03 | -1.90E-04 |

## Supplementary Figures – Monte Carlo analysis


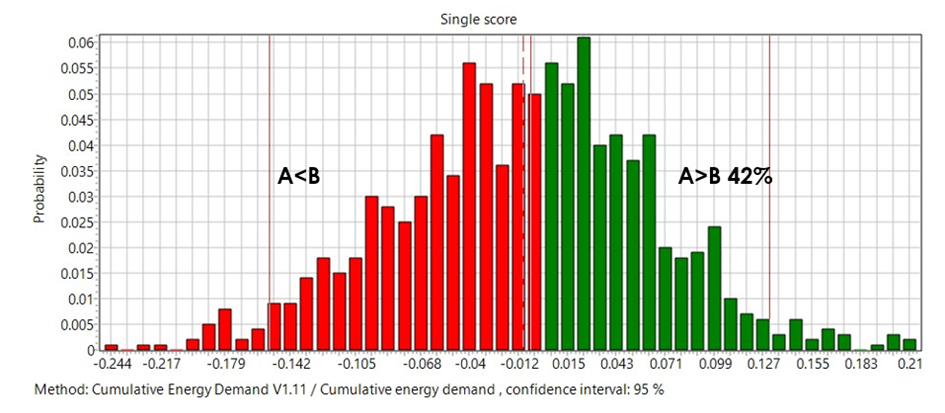


**Fig. S 1**: Monte Carlo analysis fuel cell use 4750c Scen A= (baseline, 0) H_3_PO_4_+NaOH and Scen B= (III) HNO_3_+KOH; method: Cumulative Energy Demand V1.11.


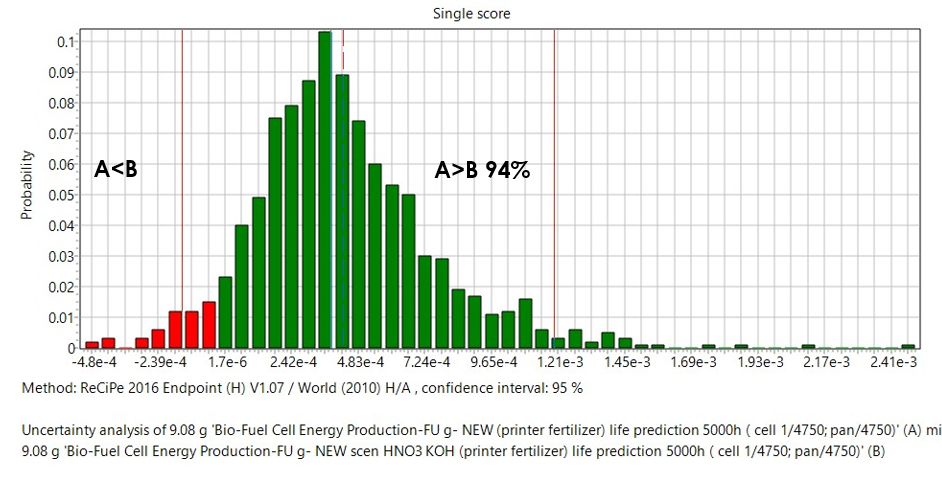


**Fig. S 2**: Monte Carlo analysis fuel cell use 4750c Scen A= (baseline, 0) H_3_PO_4_+NaOH and Scen B= (III) HNO_3_+KOH; method: ReCiPe 2016 Endpoint (H) V1.08/ World (2010) H/A.


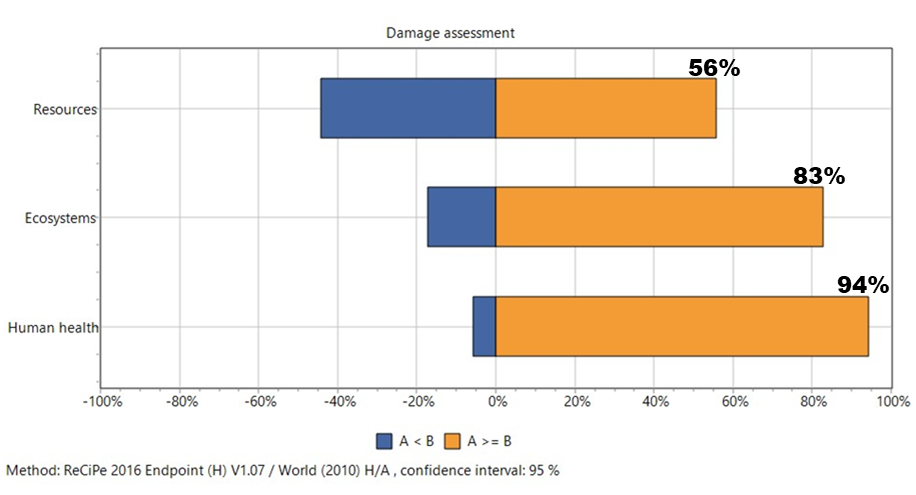


**Fig. S 3**: Monte Carlo analysis fuel cell use 4750c Scen A= *(*baseline, 0*)* H_3_PO_4_+NaOH and Scen B= (III) HNO_3_+KOH; method: ReCiPe 2016 Endpoint (H) V1.08 / World (2010) H/A, Damage assessment.

## Supplementary Figures – Energy substitution and optimization

**
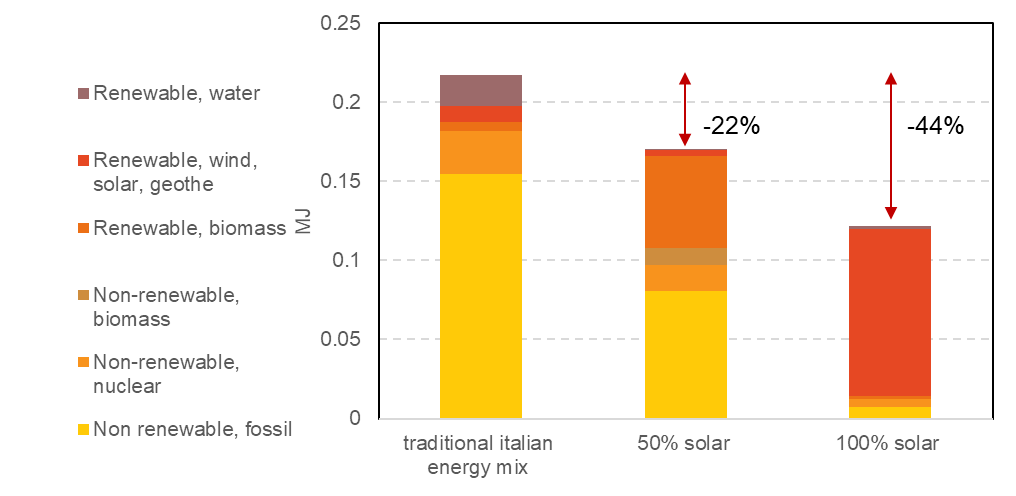
**

**Fig. S 4**: LCIA for the BFC system (FU = 9.08g treated biomass), Scenario 3 comparison of traditional Italian energy mix, 50%PV and 100%PV, Cumulative Energy Demand V1.11.


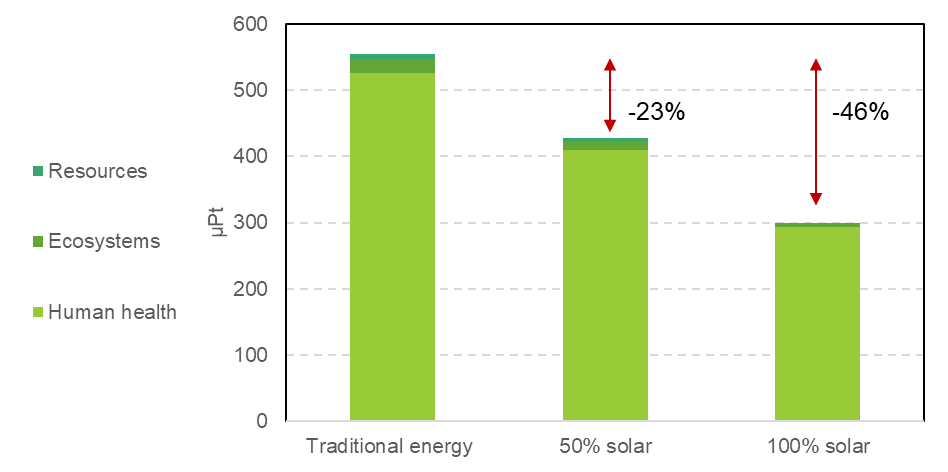


**Fig. S 5**: LCIA for the BFC system (FU = 9.08g treated biomass), Scenario 3 comparison of traditional Italian energy mix, 50%PV and 100%PV, ReCiPe 2016 Endpoint (H) V1.08 / World (2010) H/A.


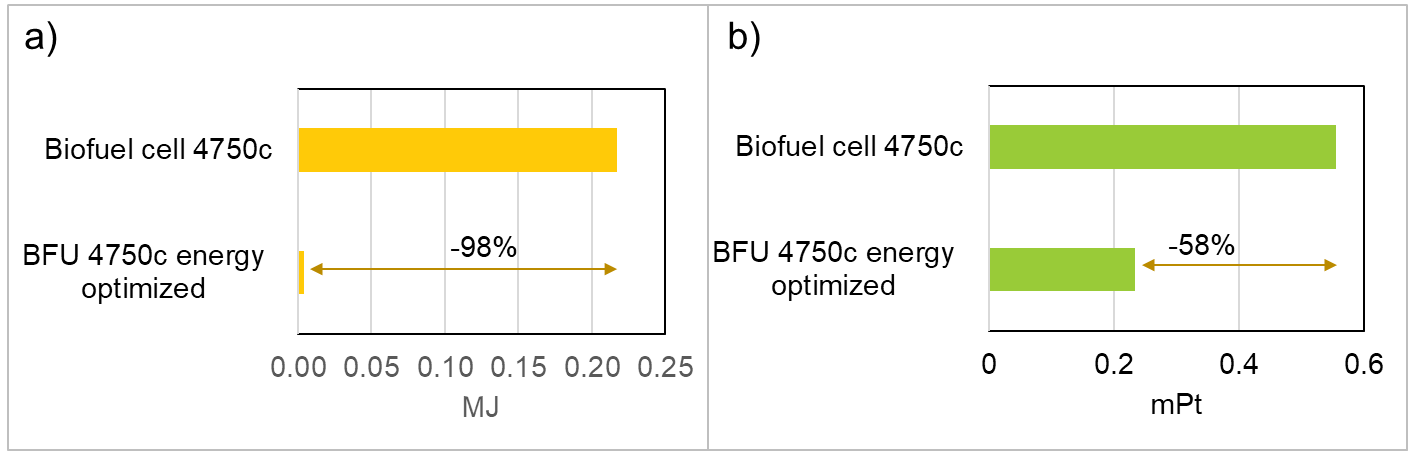


**Fig. S 6**: LCIA for the BFC system (FU = 9.08g treated biomass), Scenario 4 comparison between baseline and the energy optimized configuration in terms of a) Cumulative Energy Demand v1.11 and b) ReCiPe 2016 Endpoint (H) v1.08 / World (2010) H/A.
